# Predicting shock-induced cavitation using machine learning: implications for blast-injury models

**DOI:** 10.3389/fbioe.2024.1268314

**Published:** 2024-02-05

**Authors:** Jenny L. Marsh, Laura Zinnel, Sarah A. Bentil

**Affiliations:** ^1^ Department of Mechanical Engineering, The Bentil Group, Iowa State University, Ames, IA, United States; ^2^ Department of Mathematics, Iowa State University, Ames, IA, United States

**Keywords:** machine learning, cavitation, support vector machines, k-nearest neighbors, traumatic brain injury, shock tube

## Abstract

While cavitation has been suspected as a mechanism of blast-induced traumatic brain injury (bTBI) for a number of years, this phenomenon remains difficult to study due to the current inability to measure cavitation *in vivo*. Therefore, numerical simulations are often implemented to study cavitation in the brain and surrounding fluids after blast exposure. However, these simulations need to be validated with the results from cavitation experiments. Machine learning algorithms have not generally been applied to study blast injury or biological cavitation models. However, such algorithms have concrete measures for optimization using fewer parameters than those of finite element or fluid dynamics models. Thus, machine learning algorithms are a viable option for predicting cavitation behavior from experiments and numerical simulations. This paper compares the ability of two machine learning algorithms, *k*-nearest neighbor (*k*NN) and support vector machine (SVM), to predict shock-induced cavitation behavior. The machine learning models were trained and validated with experimental data from a three-dimensional shock tube model, and it has been shown that the algorithms could predict the number of cavitation bubbles produced at a given temperature with good accuracy. This study demonstrates the potential utility of machine learning in studying shock-induced cavitation for applications in blast injury research.

## 1 Introduction

Blast-induced traumatic brain injury (bTBI) represents over 66% of injuries sustained by deployed U.S. military service members (Regasa et al., 2019). From 2000 to the third quarter of 2022, the Department of Defense reported 486,424 traumatic brain injuries, with 387,456 of those attributed to bTBI from active deployments (DOD Worldwide TBI Numbers, 2023). bTBI is not limited to military service members but may also impact civilians in war zones or in industrial explosions. Symptoms of bTBI include visual dysfunction, headaches, balance, and impulse control impairment (Capó-Aponte et al., 2012; Bryden et al., 2019). Blast injury is also associated with an increased probability and severity of post-traumatic stress disorder (PTSD) and increased chances of developing neurodegenerative disorders (Barker et al., 2023; Borinuoluwa and Ahmed, 2023). Diagnostics, treatment, and prevention of bTBI are dependent on an understanding of the mechanisms through which blast exposure damages the brain (Marsh and Bentil, 2021).

While the process underlying bTBI remains unclear, there are several hypothesized mechanisms (e.g., thoracic surge, cavitation, and inflammation) that could improve injury models (Courtney and Courtney, 2008; Adhikari et al., 2016; Kumar Sahel et al., 2019). In the case of cavitation, which is the formation and collapse of vapor cavities in a fluid due to local pressure fluctuations, it is primarily hypothesized that the collapse of cavitation bubbles causes injury. This may be due to the pressures or temperatures generated by the bubble collapse or by the formation of high-velocity water jets, which can cause poration of cell membranes (Lafrenaye et al., 2012; Adhikari et al., 2016).

Finite element (FE) models and fluid dynamics simulations have been used in research works to examine cavitation as a mechanism of bTBI (Kurosawa et al., 2008; Panzer et al., 2012; Tan et al., 2017b). In the FE analysis of bTBI, the outcomes are dependent on a series of choices made to describe the material properties of the head in a blast exposure environment. These choices include constitutive model, geometry, and mesh properties. The constitutive model and governing equation(s) choices for materials like blood, cerebrospinal fluid (CSF), and brain tissue can differ substantially between bTBI mechanism studies (Linninger et al., 2009; Wilhelm et al., 2020; Gholampour et al., 2023). For instance, El Sayed et al. (2008) used a thermodynamic variational constitutive model that has both viscoelastic and Ogden functions. In this model, cavitation is defined by a porous plasticity term. In a study by Panzer et al. (2012), the brain tissue is modeled as a linear viscoelastic material, and the volumetric response of the CSF and brain tissue was modeled using the Mie–Grüneisen equation of state (EOS). Cavitation was modeled using the cut-off pressure method by setting a limit on the tensile pressure past a certain threshold. In a full-body blast model, Tan et al. (2017a) modeled the brain as an isotropic and viscoelastic material. Cavitation was incorporated in a CSF EOS, where the CSF density is determined by a barometric EOS, which is then used to solve for pressure in the fluid.

The geometry of the head, in the finite element model, may vary and could be as simple as cylindrical or spherical shells, with tissue and fluid surrogates inside (Kurosawa et al., 2008), to fully three-dimensional (Giudice et al., 2019; Madhukar and Ostoja-Starzewski, 2019). The head geometry can also vary by the different number of anatomical structures (e.g., white matter, gray matter, ventricles, skull, and scalp) that are included in the finite element model. For any geometry, choices about the number and size of mesh elements also have an impact on the model results (Wilhelm et al., 2020). A full-head reconstruction by El Sayed et al. (2008) contained nine structures, including the skull, CSF, and brain consisting of gray and white matter, and comprised 39,047 tetrahedral composite elements. Panzer et al. (2012) generated an axial head model with seven structures, using a single-layer mesh of 29,088 hexahedral Lagrangian elements. Some have even suggested that the inclusion of the whole body is critical in modeling blast injury and cavitation. For instance, Tan et al. (2017a) considered a full-body model that had over 4.2 million elements.

Finite element analysis is a valuable tool in understanding cavitation as a mechanism for bTBI. However, there are several limitations to FE models in bTBI research. Some of the most pressing limitations include the following: 1) All of the decisions (e.g., material properties of the head, constitutive models describing the mechanical behavior of the materials, cavitation EOS, head geometry, anatomical structure, and type and number of elements) are not standardized within the field, but directly impact the accuracy of the results of the finite element simulation of bTBI. 2) Insufficient spatial resolution (Madhukar and Ostoja-Starzewski, 2019), especially given the likely scale of cavitation bubbles (nanometers to micrometers). Thus, the spatial resolution of FE models may not be sufficient to analyze the locations and patterns of cavitation. 3) Challenges in experimental validation. There is a two-fold challenge in validating FE models of cavitation. The first is evident from the above examples: the optimal material properties and constitutive models have not been standardized. The second and a confounding issue is that experimental data would be the best way to validate these model choices, and *in vivo* evidence of shock-induced cavitation in the brain is considered implausible at this time. 4) Many finite element models use a pre-placed seed bubble, rather than modeling the actual nucleation phase of cavitation. This is because there is an ongoing debate as to whether or not there are pre-existing bubbles in the cerebrospinal fluid and blood vessels (Adhikari et al., 2016). Regardless of the debate’s outcome, including a model of cavitation nucleation is important since it impacts the biological accuracy of the results and is necessary to evaluate the ability of biological fluids to generate cavitation bubbles under realistic blast conditions.

Fluid dynamics simulations are better suited to model the fluid regions within the brain but do not perform well at modeling rigid boundary conditions and may not generate information that is easily translated to patient outcomes (Achey et al., 2022). This is partially because computational fluid dynamics (CFD) simulations have different variables than most finite element models, including wall shear stress, oscillatory shear index, and flow complexity and velocity (Achey et al., 2022). In the CFD simulations, the behaviors of the brain, CSF, and skull are generally modeled with Mie–Grüneisen or Tillotson–Brundage equations of state (Brundage, 2014; Haniff et al., 2015). However, the variation in material properties assigned to the tissue and other portions of the head is similar to that of FE models. CFD models face the same validation challenges as the FE method due to the lack of experimental data. Furthermore, CFD model accuracy may vary due to the natural variation in the head geometry, along with the volume and flow rate of CSF and blood between individuals.

There are other methods for modeling cavitation, including coupled models like fluid–structure interaction (FSI) and molecular dynamics simulations (Adhikari et al., 2016; Achey et al., 2022). These methods are valuable for the study of cavitation in blast-induced traumatic brain injury but suffer some of the same fundamental concerns as FE and CFD models: i) a lack of experimental data for validation due to the inability to visualize shock-induced cavitation *in vivo*, ii) lack of a standard set of governing equations to guide the complex model design choices, and iii) lack of nucleation (bubble formation) modeling at an appropriate spatial and temporal resolution. Thus, there is a need for alternative approaches for predicting cavitation behavior that can address some of these challenges. One such alternative approach is implementation of machine learning algorithms to classify and predict fluid behaviors like cavitation.

Machine learning algorithms show increasing potential for fluid and soft tissue modeling due to their decreased computational burden and simpler procedure, when compared to FE and CFD models (Kutz, 2017; Liang et al., 2018; Kochkov et al., 2021). Although the machine learning approach also needs to be validated against experimental data, the validation process is not as challenging when compared with the FE and CFD methods since validation of parameters describing the material properties and constitutive relations is not required. Hence, machine learning models can provide meaningful contributions to the understanding of fluid and soft tissue behavior alongside FE and CFD methods. Furthermore, machine learning algorithms can be used with integrative approaches (e.g., incorporation of heterogeneous data types and modalities) that allow for holistic and system-level understanding of biological and medical problems (Watson et al., 2019; Zitnik et al., 2019). Additionally, improved machine learning models of today can learn with less training data (e.g., data consisting of shock-induced cavitation images) than before, while yielding good to excellent accuracy (Shaikhina et al., 2015; Zhang and Ling, 2018). The smaller number of parameter choices in machine learning models helps overcome some of the challenges in experimental validation. Input and output parameter effects can be validated experimentally using a shock tube model, which is the approach taken in the present paper.

The *k*-nearest neighbor (*k*NN) and support vector machine (SVM) algorithms are two common machine learning models that have been compared in the context of both neuroscience and cavitation. Specifically, existing literature suggests that these two algorithms performed better than other algorithms in both neuroscience and cavitation applications (Feng et al., 2019; Vishwanath et al., 2020; Yakupov and Smirnov, 2023). For instance, when comparing the SVM with other machine learning techniques such as random forest and ridge regression for the purpose of cavitation prediction, the SVM showed better performance (Yakupov and Smirnov, 2023). While the actual process and parameters for each model (i.e., *k*NN and SVM) are described in the Methods section (Section 2), a brief description of the algorithm’s prior use in neuroscience and cavitation research is presented here.

Within the field of neuroscience, *k*NN and SVM have both been used and compared to classify brain injury and other neurological outcomes (e.g., survival, diagnostic scores, and functional connectivity) using electroencephalography (EEG) and magnetic resonance imaging (MRI) data (Vergara et al., 2017; 2018; Hale et al., 2018). When using EEG data to classify mild traumatic brain injury (mTBI), a *k*NN model with three neighbors outperformed the SVM, although the two models were usually within 3% of each other (Vishwanath et al., 2020). The *k*NN and SVM algorithms have also been applied to patient clinical data. For example, the SVM has previously been shown to perform better than logistic regression in classifying survival rates in severe traumatic brain injury (sTBI) (Feng et al., 2019). A study by Hsu et al. (2021) found that both *k*NN and SVM did comparably well in predicting patient survival following sTBI, using clinical data, but were much less successful at accurately classifying patient death.

*k*NN and SVM have also been used in cavitation models (Fadaei Kermani et al., 2018; Dutta et al., 2020). For instance, a *k*NN model was able to accurately predict the severity of cavitation damage on a dam spillway during periods of flooding (Fadaei Kermani et al., 2018). Additionally, a comparative study of *k*NN and SVM has also been performed by Dutta et al. (2020) to detect cavitation in a pumping system. The results showed that *k*NN is preferable when there are more training data than features (i.e., variables), while the SVM is better at classifying larger amounts of labeled data. The labeled data in the study by Dutta et al. (2020) were variables with assigned values, where these values described the centrifugal pumping system (e.g., cavitation status and rotational speed).

There is limited literature applying machine learning to detect and predict cavitation bubbles in biological and neuroscience applications. As such, this paper presents an example of how machine learning can predict fluid behavior (i.e., cavitation) as a function of a fluid property that is relevant in a biological system (i.e., temperature). Experimental images of shock-induced cavitation, in fluids at different temperatures, were recorded during shock tube experiments. The images were fed into a bubble detection program to generate datasets quantifying the number of cavitation bubbles as a function of temperature. These datasets were used to train two commonly used machine learning algorithms (i.e., *k*NN and an adaptation of the SVM that will facilitate multi-class classification) in a comparative study to understand which algorithm could best predict the cavitation level (i.e., number of cavitation bubbles) given the temperature of the surrounding fluid. Thus, the novelty of this paper is in using the *k*NN and SVM algorithms to predict shock-induced cavitation behavior based on a biological parameter (i.e., temperature). Additionally, the results from the machine learning models were validated with experimental data that visualized shock-induced cavitation. The findings from this paper will influence future experiments investigating cavitation as a bTBI mechanism by demonstrating the ability of machine learning algorithms to predict cavitation behavior without a defined set of governing equations or model properties.

In the future, the machine learning model presented in this study will be expanded such that multiple inputs (e.g., geometry, temperature, and blast wave pressure) can be considered to predict the corresponding fluid behavior (i.e., cavitation). Such a model could then be adapted in the manner similar to Fadaei Kermani et al. (2018), where the level of cavitation predicted can be translated into a prediction of damage level (in this case bTBI severity).

The remainder of this paper presents a description of the bubble detection and bubble classification algorithms used in the shock-induced cavitation study. Furthermore, the performance of the *k*NN and adapted SVM models at predicting shock-induced cavitation is provided. The discussion in Section 4 evaluates the selection of the ideal machine learning algorithm for modeling fluid behavior in bTBI and includes suggestions for expanding cavitation-specific machine learning models in the future.

## 2 Methods

### 2.1 Cavitation chamber

**FIGURE 1:**
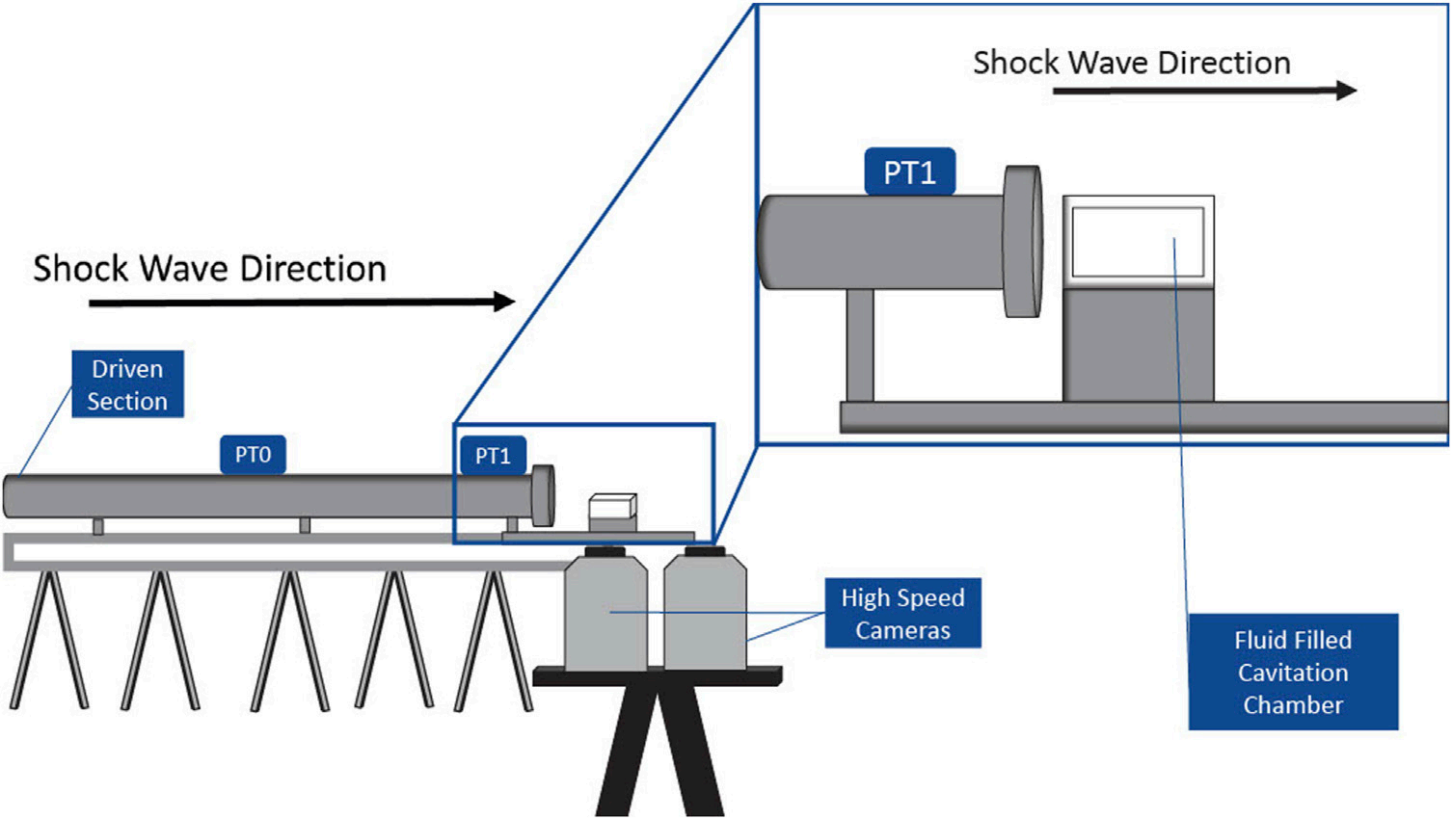
Experimental placement of the cavitation chamber, shock tube, and high-speed cameras.

A 50 mm × 25 mm × 25 mm cavitation chamber was constructed of clear, acrylic sheets for this study. Acrylic sheets were selected not only for durability but also for optical clarity to facilitate imaging of the shock-induced cavitation event (described in Section 2.2). A depiction of the experimental setup, including the cavitation chamber, shock tube, pressure transducers, and cameras, is shown in Figure 1. The wall of the cavitation chamber that is in front of the shock tube was the thinnest (1 mm). The side walls were 5 mm thick, and all the remaining walls were 10 mm thick.

The cavitation chamber was filled with 31 mL of deionized water by using a syringe. Deionized water was used to avoid the residual effect of any ions or electrical conductance on cavitation.

The deionized water was heated to the desired temperature by using a hot plate, and the temperature was recorded with a graduated tube thermometer and a digital thermometer function (Traceable Salinity Pen, model 4367). Five trials were used for each testing temperature, which ranged from 20°C to 60°C in 5°C increments.

The fluid temperature was recorded before and after filling the chamber and after shock exposure. In between shock exposures, the deionized water was removed from the chamber by using a syringe.

### 2.2 Shock tube model

A three-dimensional (3-D) shock tube model was used to generate the shock waves, which induced cavitation in chambers filled with deionized water. The 76.2-mm oxyacetylene shock tube is divided into a driver (0.3 m) and a driven (4.6 m) section, separated by a 25.4-μm Mylar diaphragm. The oxyacetylene in the driver section was ignited, which ruptured the diaphragm and generated a shock wave that propagated down the driven section of the shock tube and toward the cavitation chamber. The fluid-filled cavitation chamber was placed 2 mm from the exit of the shock tube. Pressure transducers (PTs) record the speed and pressure–time profile of the shock wave. Piezoelectric pressure transducers 0 and 1 (PT0 and PT1, PCB Piezotronics, Model 102B15) are 1.5 m apart and are located on the driven section of the shock tube so that the shock wave speed and overpressure can be measured (see Figure 1). PT1 is 127 mm from the front wall of the cavitation chamber. The cavitation chamber was illuminated using two separate LED lights (Nila Zaila Deluxe Daylight) prior to igniting the oxyacetylene in the shock tube.

During each trial of the shock tube experiments, the images of the cavitation chamber were recorded at 100,000 frames per second using two high-speed monochrome digital cameras (Photron, FASTCAM SA-Z) with a 105-mm macro lens (Nikon, AF-S VR Micro-NIKKOR 105-mm f/2.8G IF-ED). In this study, a trial refers to one ignition of the oxyacetylene in the shock tube and the subsequent recording of the cavitation event (or lack thereof, if cavitation did not occur). A subset of 50 images, which covers 0.5 ms, was found to completely depict the cavitation event. This subset of 50 images was saved for each trial, resulting in a total of 2,250 images for the 45 trials conducted since there were five trials at each 5°C increment. These 2,250 images were processed using the bubble detection program described in Section 2.3.

### 2.3 Bubble detection program

**FIGURE 2:**
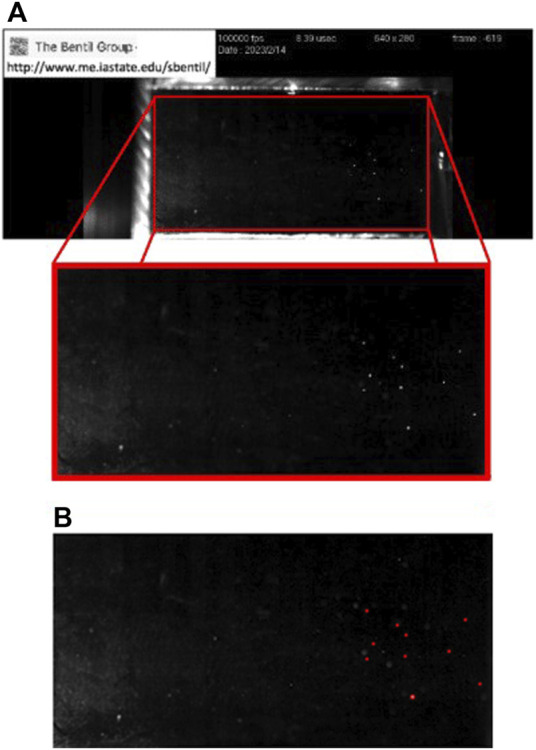
Cavitation bubble detection process using an image from a trial at 60°C. **(A)** The cavitation region of interest consists of the entirety of the chamber interior (red rectangle), which is cropped for use in the bubble detection program. **(B)** Example of the output from the bubble detection program for the selected region of interest. The annotated image is created by the bubble detection program, where the detected bubbles are outlined in red.

A bubble detection program was written using the commercially available software MATLAB (version: 9.10.0.1602886 (R2023a)). The program detected the bubbles automatically within the entirety of the chamber’s interior, which was selected as the cavitation region of interest. The region of interest for the first image in the series was selected manually, and all subsequent images were batch-cropped to the same region. An example of image cropping, as well as bubble detection for the region of interest, is shown in Figure 2.

Each cropped image was then pre-processed to minimize noise by removing artifacts, such as light reflections from the chamber’s exterior or shock tube. The MATLAB function “imfindcircles” was used to detect the bubbles in each processed image. The center coordinates were recorded for all of the bubbles in each of the images. A count of the number of bubbles (i.e., bubble count) and bubble locations was recorded for each image. Each bubble was assigned an identification number based on its first appearance to facilitate tracking bubbles through the frames recorded by using high-speed cameras (Crocker, 1999). This allowed individual cavitation bubbles in the image series to be counted, without repeatedly counting bubbles which occur in multiple frames. At each 5°C increment, between 20°C and 60°C, the data from each image describing the total bubble counts and bubble locations were recorded. The grand total number of shock-induced cavitation bubbles and bubble locations used in each trial was obtained by aggregating the total bubble counts and bubble locations from all the images in an image series.

### 2.4 Machine learning algorithms

A *k*-nearest neighbor (*k*NN) model and an adapted support vector machine model using error-correcting output codes (ECOC SVM) were constructed to predict shock-induced cavitation behavior as a function of temperature. The *k*NN and ECOC SVM machine learning models use fluid temperature as an input and the level of cavitation (i.e., cavitation level) as an output. The cavitation level refers to the grand total number of shock-induced cavitation bubbles produced at a specified fluid temperature, which is described further in Section 2.5. Fluid temperature refers to the bulk temperature of the deionized water that is inside the cavitation chamber.

Both *k*NN and SVM are supervised machine learning methods, meaning they use labeled points with known solutions to train the machine learning models. As a result, the machine learning models are first trained to predict the cavitation level using labeled training points, where each “point” represents one experimental trial. The training points are considered “labeled” because the *k*NN and ECOC SVM machine learning models have been provided with both the input (i.e., fluid temperature) and the output (i.e., level of cavitation) for each trial. After training the *k*NN and ECOC SVM models using the labeled training points, the machine learning models are able to classify cavitation levels using testing points. A testing point is an experimental trial at a given fluid temperature, which is given as an input for the machine learning model to predict the cavitation level. As such, the output from using a testing point in the *k*NN and ECOC SVM models is a prediction of the cavitation level given a fluid temperature. Thus, the machine learning model’s predictive accuracy can be measured by comparing the predicted level of cavitation for a given testing point (i.e., an experimental trial) with the actual cavitation level calculated using the bubble detection program from the experimental trial.

**FIGURE 3:**
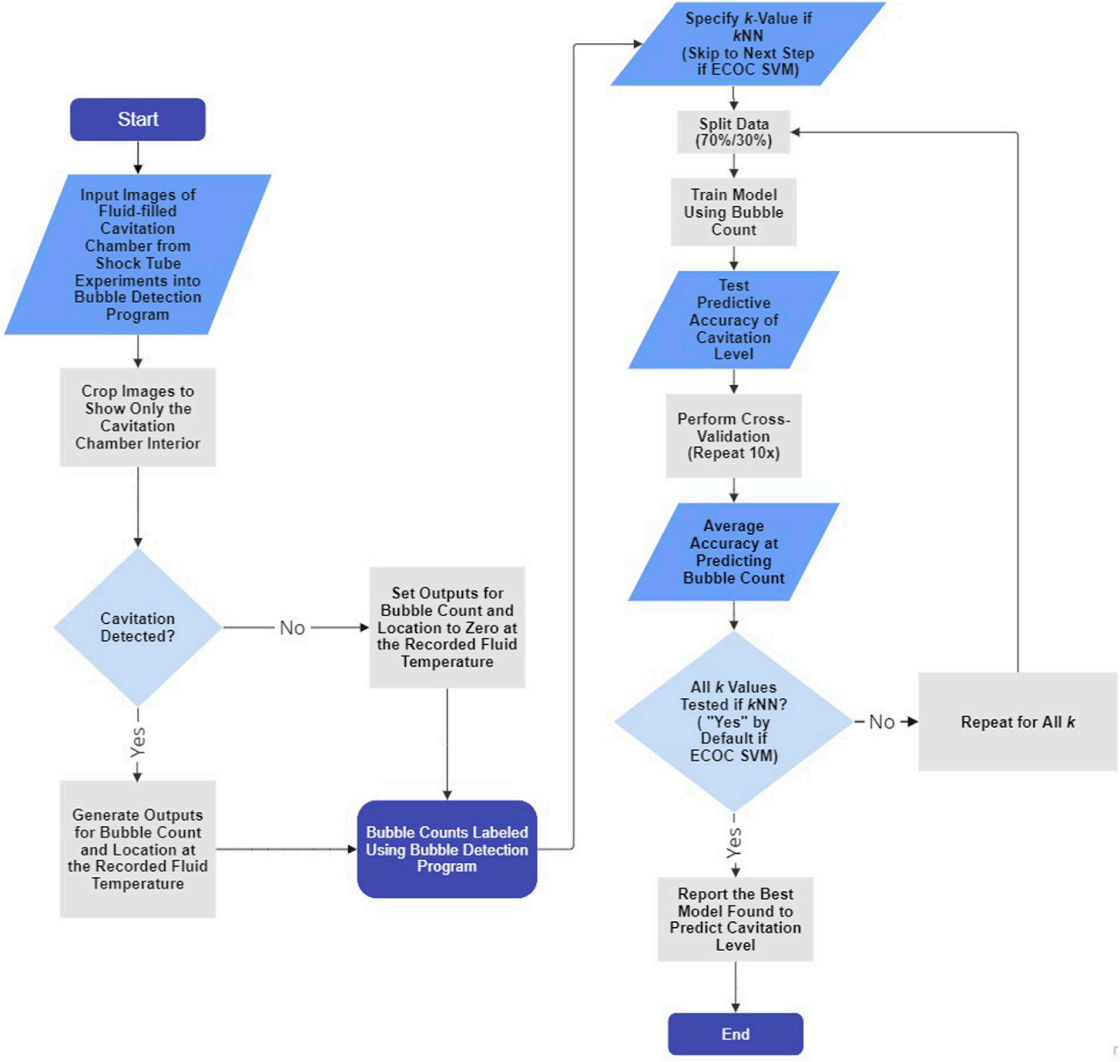
Flowchart depicting the bubble detection program and machine learning algorithm processes for each trial. Steps that do not apply to ECOC SVM and only apply to *k*NN are indicated. The *k*NN and ECOC SVM algorithms described using the flowchart require one input (i.e., fluid temperature) and yield one output (i.e., cavitation level). The flowchart was generated using the free version of Miro Mir (2023).

Figure 3 illustrates the coupled bubble detection and machine learning (i.e., *k*NN and ECOC SVM) processes.

#### 2.4.1 *k*NN algorithm

The *k*NN algorithm is a simple machine learning method that classifies the output from a testing point based on the categories (i.e., known outputs/solutions) of labeled training points (i.e., nearest neighbors) (Zhang, 2016).

The output from a testing point is the category that contains the highest number of the *k*-labeled nearest neighbors (i.e., labeled training points that had a similar output). In the context of this study, the testing point is a fluid temperature for which the machine learning model is not given the level of cavitation. The machine learning model predicts the cavitation level for the testing point by using the labeled training data, where both the input (i.e., fluid temperature) and output (i.e., cavitation level) are provided to the machine learning model.

The number of *k* neighbors used to determine the category of a testing point can affect the performance of the model. A *k*-value that is too large can lead to underfitting, while a *k*-value that is too small can lead to overfitting (Zhang, 2016). Here, underfitting means the model is ignoring important details and patterns (e.g., increases or decreases in the cavitation level with respect to temperature) within the data, and overfitting means that the model is learning too many small details (noise) from the training data such that the model’s predictions are not generalizable.

The commonly suggested value for *k* is the square-root of the number of training samples (Zhang, 2016).

This suggests that a *k*-value of approximately 5 would be appropriate for these data. However, five separate *k*NN models were trained using *k* values of 1, 2, 3, 5, and 7 to determine whether the suggested *k* was the optimal value. The values considered for *k* were predominantly odd because even values of *k* historically have poor classification ability (Pawlovsky and Kurematsu, 2019).

#### 2.4.2 *k*NN cross-validation

Cross-validation is a common way of testing potential *k*-values to determine the best choice of *k* for a given dataset (Biessey et al., 2021). The optimal *k*-value was determined through cross-validation using an adapted holdout technique. The adapted holdout technique was used for the cross-validation because it helps avoid bias in the reported accuracy by taking an average of the accuracies of models trained on different train–test splits (Biessey et al., 2021). A *k*-value set to the square root of the trial number would in this case be approximately 5, which is why 5 was included as a possible *k*-value. However, this is only a standard recommendation (Zhang, 2016).

For each *k*-value (i.e., 1, 2, 3, 5, and 7), the cross-validation required the creation of 10 different *k*NN models using the fitcknn function in MATLAB.

The purpose of this cross-validation process is to avoid biased results.

Each of the 10 *k*NN models, for a specific *k*-value, were trained and tested using a different train–test split with 70% training points and 30% testing points. While there is no universally agreed upon ideal train–test split ratio, the testing set needed to be of sufficient size to evaluate the performance of the model. The structure and size of the data favored the selection of a 70%/30% split over a split of 80%/20% to be consistent with related literature (Hale et al., 2018). This is because the 70%/30% split is the standard recommendation for smaller datasets since higher splits are reserved for large datasets (Muraina, 2022).

The MATLAB function cvpartition was used to randomly split the data into 70% training and 30% testing sets. The machine learning model was trained using the training set, and the function crossval was used to calculate the accuracy of the model in predicting the cavitation level on the testing set. This process was repeated 10 times using different train–test sets for a specific *k*-value, and the testing accuracies were averaged to obtain the cross-validation accuracy.

The training and testing data both contained the fluid temperature and actual cavitation level of each point (i.e., experimental trial). However, the *k*NN is not provided with the actual cavitation level when using the testing set to make its prediction of the grand total number of cavitation bubbles as a function of temperature. Instead, the actual cavitation level associated with the testing point is used to assess the accuracy of the *k*NN’s prediction.

The *k*NN model requires the distance between the labeled points (from the training dataset) to be defined. This distance affects which labeled points are chosen as the nearest neighbors to a testing point. The Euclidean distance was chosen for the *k*NN model due to the simplicity of having only a single input (i.e., temperature) to predict the cavitation level (Zhang, 2016). Thus, the Euclidean distance *d* between the test point with temperature *x* and a neighbor (i.e., labeled point) with temperature *y* is *d*(*x*, *y*) = |*x* − *y*|.

#### 2.4.3 ECOC SVM algorithm and cross-validation

An SVM is a machine learning model used for binary classification tasks. The SVM model finds the hyperplane separating two groups of labeled points, which maximizes the margin between the hyperplane and the nearest labeled data point in each class (Guenther and Schonlau, 2016). Since the objective of predicting the various cavitation levels as a function of fluid temperature is not a binary classification task, the SVM is adapted using the ECOC. The ECOC modification enables the SVM model to be generalized to solve multi-class classification problems by combining several SVMs that are each trained to perform a different binary classification task (Yan and Yang, 2014). The ECOC SVM models were created in MATLAB using the fitcecoc function, and the ECOC SVM models were cross-validated in the same manner as the *k*NN models (Section 2.4.2).

Specifically, the data were randomly split into 70% training and 30% testing sets using the MATLAB function cvpartition. After the ECOC SVM model was trained on the training set, the function crossval was used to calculate the accuracy of the model in predicting the cavitation level using the testing set. This process was repeated 10 times, and the testing accuracies were averaged to obtain the cross-validation accuracy.

#### 2.4.4 Confidence intervals from the bootstrapping method

To further validate our model performance results, a bootstrapping method was used to obtain confidence intervals for the average accuracy of all *k*NN and ECOC SVM models. Bootstrapping methods are used to approximate the mean of a distribution by taking the means of several random samples and using the sample mean distribution to determine a confidence interval for the true mean (Wehrens et al., 2000). This confidence interval is more informative than a single sample mean because it uses the standard deviation of the sample means to give a range of values that contain the true mean with some level of confidence (i.e., 95% confidence).

For each model (i.e., *k*NN or ECOC SVM with a given cavitation scheme), a sample of 10 trained models was collected by training each one on a different random 70%/30% train–test split. The test accuracies of all 10 models were averaged to obtain a sample mean (i.e., the mean accuracy for that sample of 10 trained models). This process was repeated 10 times to obtain a set of 10 sample means. Finally, the average and standard deviation of the set of 10 sample means were used to construct a 95% confidence interval for the average accuracy of the given model. This entire bootstrapping process was repeated for all 24 models.

### 2.5 Defining cavitation level

Cavitation level is not something that has been previously defined, although prior studies have applied levels to cavitation damage (Fadaei Kermani et al., 2018). Thus, this manuscript defines the cavitation level as the number *n* of cavitation bubbles that appear after the cavitation chamber is exposed to a shock wave with an overpressure of 207 kPa from the pressure transducer PT0. The different levels of cavitation are described using four different “cavitation schemes.” These four schemes assess how precisely the *k*NN and ECOC SVM models could predict cavitation bubble numbers (e.g., delineating 5–10 bubbles from 15–20 bubbles) as a function of temperature.

Since a standard definition for the “cavitation scheme” does not exist, two different approaches were considered to determine the appropriate bin sizes at each cavitation level. The first approach is “data-driven” because bins are selected based on the distribution of the cavitation bubbles obtained from the shock tube experiments. The second approach is “distribution-driven” and focuses on the similarity of bin sizes, regardless of the actual distribution of the cavitation bubbles from the shock tube experiments. Two different cavitation schemes were explored for both the “data-driven” and “distribution-driven” approaches.

Table 1 shows the definition of each cavitation level for the two different approaches and four cavitation schemes. These cavitation level definitions apply to both the *k*NN and ECOC SVM models.

**TABLE 1:** Definitions of the cavitation level by bubble number *n* for four different cavitation schemes and two approaches.

	Data-driven approach		Distribution-driven approach	
	Scheme 1	Scheme 2	Scheme 3	Scheme 4
Cavitation level 1	*n* ≤ 5	*n* ≤ 1	*n* ≤ 1	*n* ≤ 1
Cavitation level 2	5 < *n* ≤ 20	1 < *n* ≤ 5	1 < *n* ≤ 5	1 < *n* ≤ 5
Cavitation level 3	*n* > 20	5 < *n* ≤ 10	5 < *n* ≤ 10	5 < *n* ≤ 10
Cavitation level 4		10 < *n* ≤ 25	10 < *n* ≤ 25	10 < *n* ≤ 15
Cavitation level 5		25 < *n* ≤ 75	25 < *n* ≤ 50	15 < *n* ≤ 20
Cavitation level 6		*n* > 75	50 < *n* ≤ 100	20 < *n* ≤ 30
Cavitation level 7			*n* > 100	30 < *n* ≤ 40
Cavitation level 8				40 < *n* ≤ 50
Cavitation level 9				*n* > 50

The two cavitation schemes (i.e., schemes 1 and 2) for the “data-driven” approach were selected to reflect the actual numbers of cavitation bubbles observed in each trial. This means that the levels were defined such that each level reflected the actual cavitation bubble number of at least one trial, and there were no levels that did not correspond to a real trial. For example, if the trials contained 1, 2, 2, 5, 8, 10, and 12 bubbles, then the cavitation levels might be 1–5 and 8–12. The cavitation levels are not necessarily equally sized and are selected such that each level will have training trials that fall within those cavitation levels.

The two cavitation schemes (i.e., schemes 3 and 4) for the “distribution-driven” approach were created using equally sized bins. These equally sized bins may contain cavitation levels that are not associated with a trial since the number of cavitation bubbles at that level may not have been observed during the shock tube experiments.

## 3 Results

### 3.1 Shock wave pressures

**FIGURE 4:**
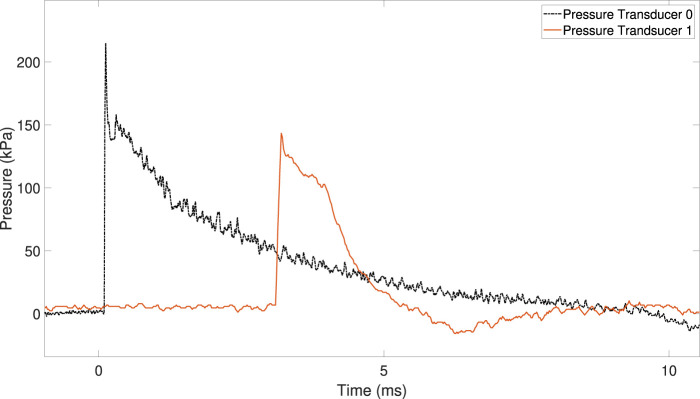
Pressure–time traces from pressure transducers PT0 and PT1, located on the driven section of the shock tube.

Figure 4 shows the pressure history for PT0 and PT1 during the shock tube experiments. The shock wave speed inside the shock tube was 484 m/s (Mach 1.4).

The overpressure recorded by PT0 and PT1 during the experiments was 207 kPa and 148 kPa, respectively.

The magnitude and duration of the overpressure decrease when the shock wave approaches the shock tube exit (i.e., the end of the driven section).

### 3.2 Bubble detection program

Using the recorded images from the shock tube experiments, the bubble detection program produced the grand total number of shock-induced cavitation bubbles detected as well as an assigned identification number for each bubble based on its first appearance (and location) to facilitate tracking of bubbles through the frames. To ensure accurate performance of the bubble detection program, the bubble number was manually counted from a subset of 75 randomly selected images where shock-induced cavitation was present. A subset size of 75 was chosen to validate the bubble detection program, which corresponds to 10% of the images from trials containing shock-induced cavitation. The variance between the bubbles counted manually and those counted using the bubble detection program did not exceed five (5) bubbles, even at the highest cavitation levels.

The performance of the bubble detection program at different temperatures is depicted in Table 2.

**TABLE 2:** Mean number of bubbles following the manual count and using the bubble detection program as a function of fluid temperature. The fluid temperature is the input parameter for the machine learning model. The last column is the difference in the mean number of bubbles calculated using the formula: manual–program.

	Mean number of cavitation bubbles		
	Manual	Program	Difference
20°C	0.32	0.12	+0.2
30°C	0.86	0.71	+0.15
40°C	4	6.4	−2.4
50°C	22.3	28	−5.7
60°C	92	97.5	−5.5

In addition, the bubble detection program also saves an annotated image consisting of cavitation bubbles that are outlined using the color red. An example of this bubble detection annotation is shown in Figure 2B for the 60°C case.

### 3.3 *k*NN performance as a function of *k* choice and cavitation scheme

The following subsections describe the performance of the *k*NN algorithm, which varied by the choice of *k* and cavitation scheme.

#### 3.3.1 *k* choice

The grand total of the number of bubbles was used to train the *k*NN models using a *k*-value of 1, 2, 3, 5, and 7 neighbors to facilitate prediction of the cavitation level.

The optimal *k*-values were determined based on the cross-validation model accuracy, which is the average accuracy of predicting the cavitation level using ten *k*NN or ECOC SVM models, where each machine learning model was trained and tested on a new random train–test split of 70% training and 30% testing, as described in Section 2.4.

**FIGURE 5:**
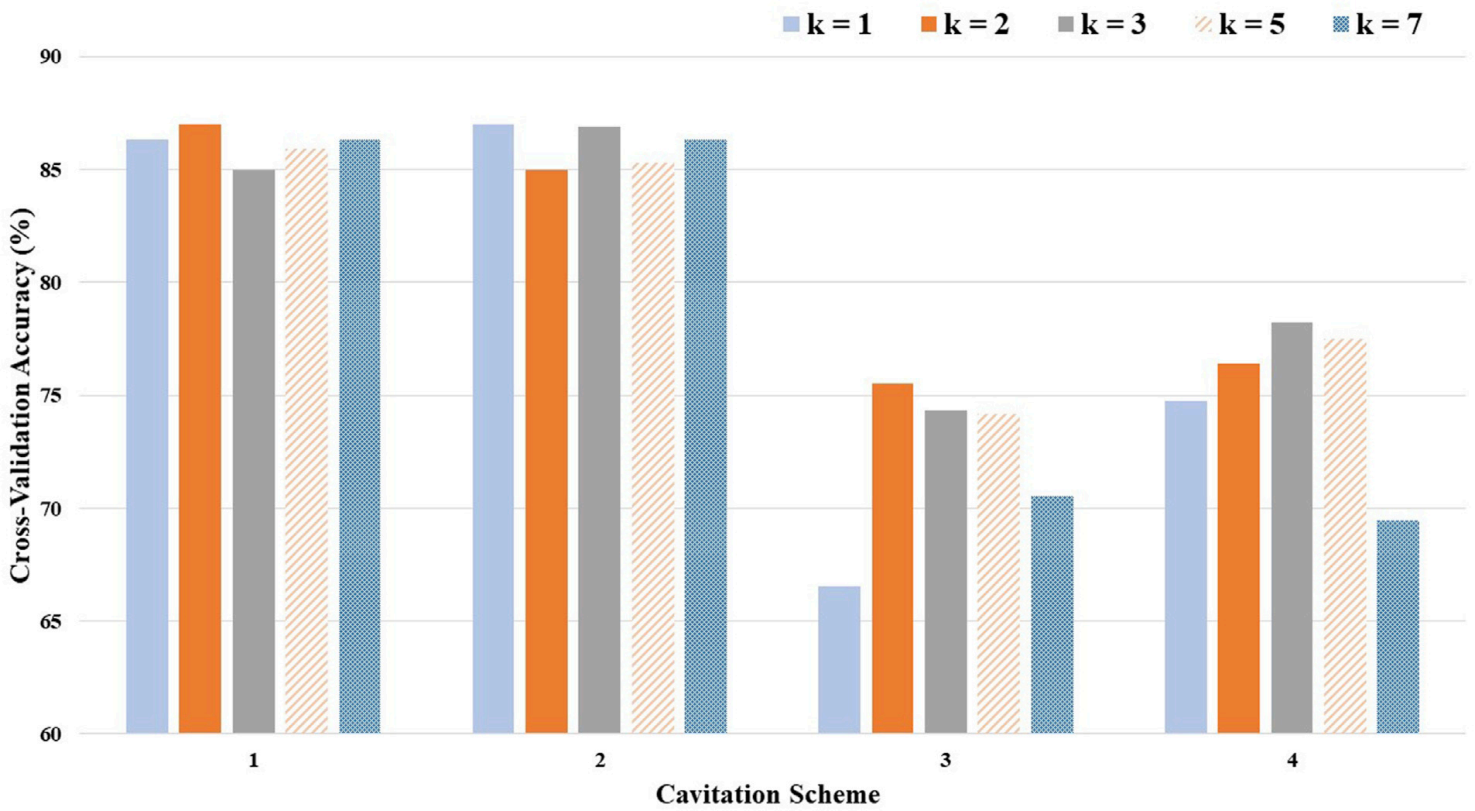
Cross-validation accuracy by the choice of *k* and the cavitation scheme.

The optimal (i.e., highest performing) value for *k* depended on the levels defined by the cavitation scheme (Table 1). The cross-validation accuracies, by choice of *k* and cavitation scheme, are depicted in Figure 5.

Considering the average cross-validation accuracy across all four cavitation schemes, a *k*-value of 3 performed the best, while the recommended *k*-value of 5 performed only slightly worse (1% difference). However, the optimal *k*-value also varied by the cavitation scheme. For example, a *k*-value of 1 produced the best results for cavitation scheme 2, but the worst results for cavitation scheme 3.

**FIGURE 6:**
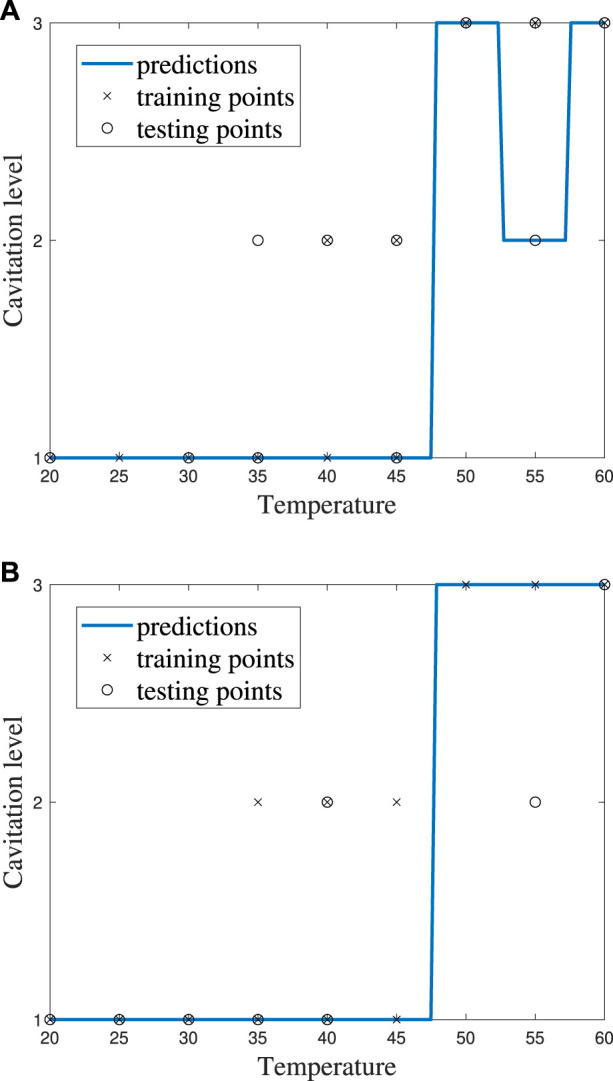
*k*NN model performance using cavitation scheme 1 for **(A)** *k* = 2, which is the best performing value of *k*, and **(B)** *k* = 3, which is the worst performing value of *k*.

**FIGURE 7:**
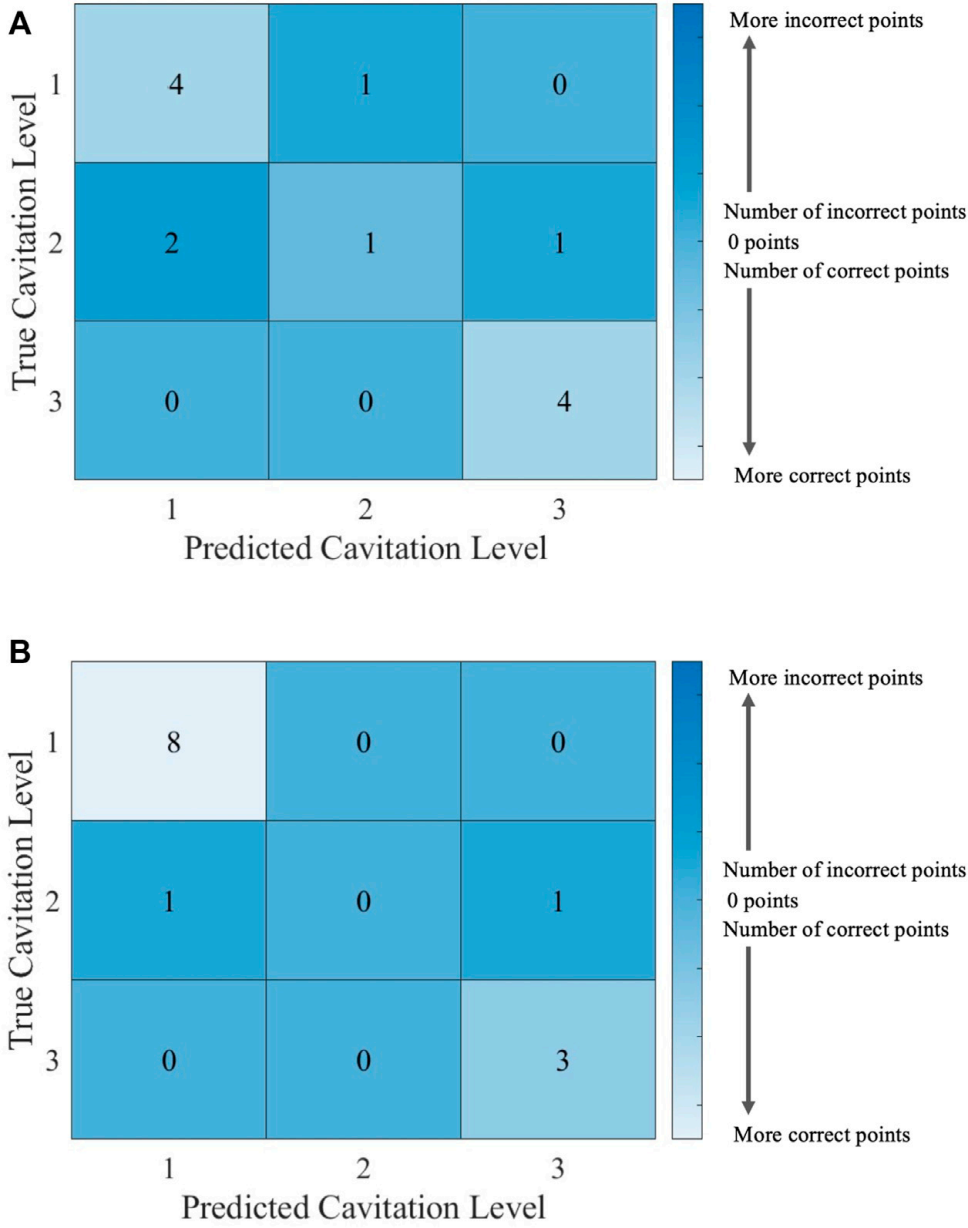
Confusion matrices for the *k*NN model using cavitation scheme 1 with **(A)** *k* = 2, which is the best performing value of *k*, and **(B)** *k* = 3, which is the worst performing value of *k*. The total number of rows and columns in the confusion matrix correspond to the number of cavitation levels defined by the cavitation schemes in Table 1.

**FIGURE 8:**
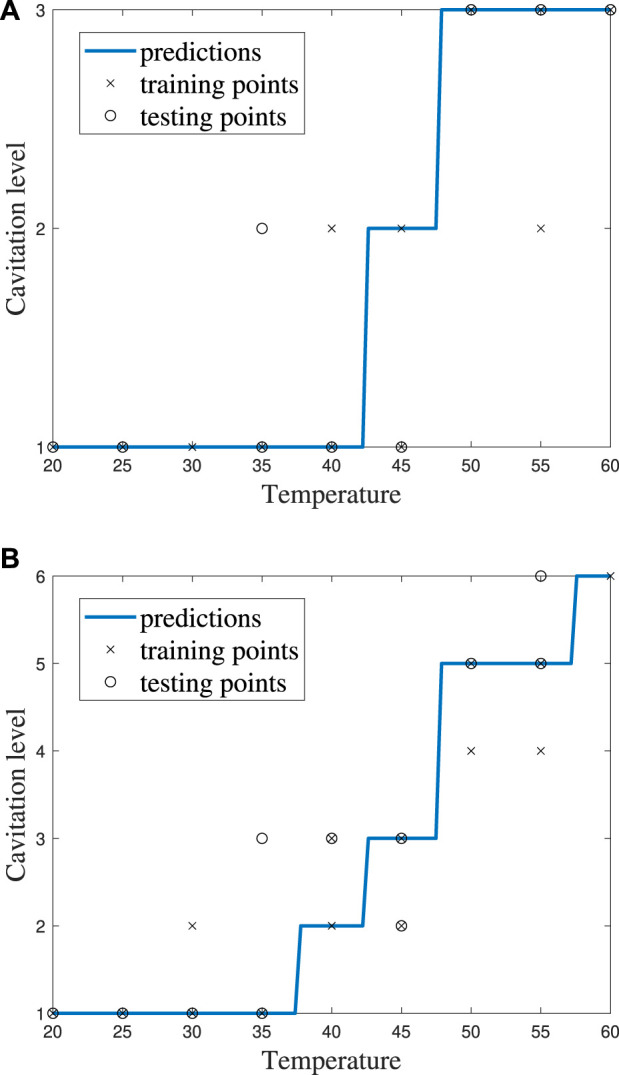
*k*NN model performance for *k* = 5 using **(A)** cavitation scheme 1 and **(B)** cavitation scheme 2.

**FIGURE 9:**
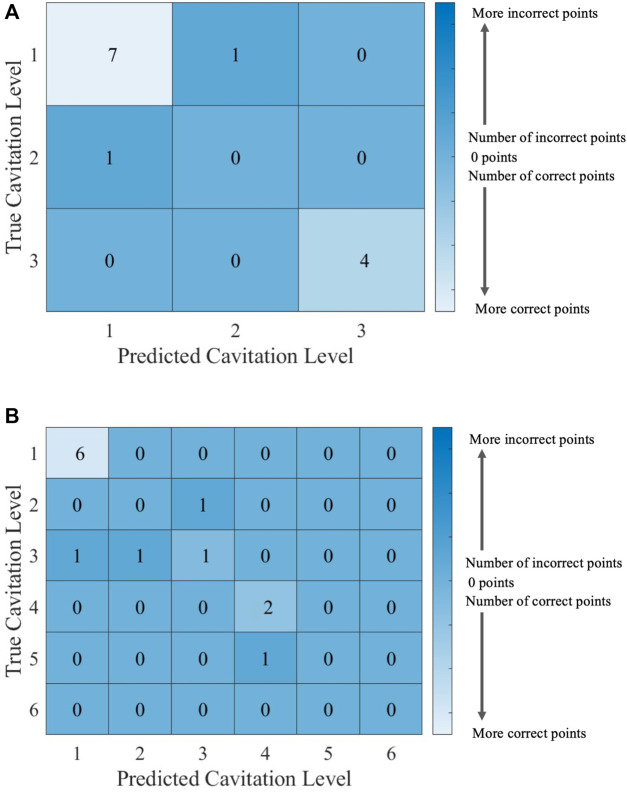
Confusion matrices for the *k*NN model with *k* = 5 using **(A)** cavitation scheme 1 and **(B)** cavitation scheme 2. The total number of rows and columns in the confusion matrix correspond to the number of cavitation levels defined by the cavitation schemes in Table 1.

It can be helpful to compare the results of an individual machine learning model to gain more insights into where predictions were correct and incorrect. Individual model accuracy was depicted using a graphical representation (i.e., Figure 6; Figure 8), where a prediction line is used to illustrate when the *k*NN model predicted the testing points. In the graphical representation, the predictive outputs for the cavitation level at the different values of *k* are shown. Confusion matrices for the predictions by the *k*NN model are also provided (i.e., Figure 7; Figure 9). In the confusion matrix, the rows describe the true class and the columns represent the predicted class. The true class is the actual cavitation level corresponding to the testing point, and the predicted class is the cavitation level predicted by the machine learning model for that testing point’s temperature. The numbered rows and columns in the confusion matrix correspond to the cavitation level in a scheme. For example, a confusion matrix for cavitation scheme 1 would be 3 × 3 since there are three possible cavitation levels. The degree of correctly classified points in the legend and elements of the confusion matrix describes the number of testing points that were correctly classified by the model.

Since there are 13 testing points that are randomly selected, following the 70% training/30% testing split of the data, the sum of all the values in each confusion matrix is 13. Thus, the confusion matrix provides the outcome for each of the 13 testing points considered. There may be more than one training and testing point at each temperature for a given cavitation level and scheme. This is attributed to the randomly generated 70% training/30% testing split of the data. The plots of *k*NN performance (Figure 6; Figure 8) do not show the number of testing and training points at a given temperature. Furthermore, the confusion matrices (Figure 7; Figure 9) for the *k*NN performance highlight the number of testing points at a cavitation level, but do not indicate the number of points occurring at a specific temperature. As a result, the number of testing and training points at a given temperature for the different *k*NN models is included in Supplementary Tables S1–S4, which can be found in Supplementary Materials.

#### 3.3.2 Cavitation scheme

The cavitation scheme had a substantial impact on model performance. While there was not a large difference between the two data-driven schemes (1 and 2) or between the two distribution-driven schemes (3 and 4), the variation in performance between the data- and distribution-driven schemes was large. The data-driven schemes performed 10%–20% better than the distribution-driven schemes, regardless of the *k*-value. It is worth noting that even the worst performing *k*-value (i.e., *k* of 1) in the worst performing scheme (i.e., scheme 3) still achieved an accuracy of 66.54%.

The choice of the cavitation scheme impacts the cavitation level predicted by the *k*NN model. For example, Figure 8 shows the model performance for a *k*-value of 5 using schemes 1 and 2, while the confusion matrices for these two schemes are shown in Figure 9. Figure 8A shows that the model does not predict cavitation level 2 well using cavitation scheme 1 (5 < *n* ≤ 20 bubbles) since the prediction (blue line) made by the model passes through only one classified testing point (i.e., a fluid temperature of 45°C). Comparatively, Figure 8A shows that the model predicts cavitation levels 1 and 3 well when considering scheme 1 since the prediction line passed through all of those classified testing points. The confusion matrix for the results in Figure 8A is shown in Figure 9A, where the number of testing points that were predicted correctly and incorrectly by the model is shown. Additionally, Figure 8B shows that the model does not do as well at predicting cavitation levels 2–4 using cavitation scheme 2. As a result, it can be discerned that the machine learning model is not accurately distinguishing whether a given temperature will produce 5–10 cavitation bubbles by comparing the cavitation level definitions given in Table 1. This is because 5–10 cavitation bubbles can occur at several different temperatures (e.g., 35°C, 40°C, and 45°C).

### 3.4 ECOC SVM performance as a function of the cavitation scheme

The ECOC SVM outperformed the *k*NN model for all the cavitation schemes considered. This may be due to the lack of a parameter, similar to the *k* choice for the *k*NN, that needs to be optimized for the ECOC SVM model. The ECOC SVM model does require a “cost” parameter *C*, which affects the weight (or cost) assigned by the algorithm to each misclassified training point (i.e., the labeled point containing the fluid temperature and the actual cavitation level information from the trial). However, *C* is automatically processed using soft-margin minimization by MATLAB, which assigns the cost of each misclassification of the cavitation level based on the distance between the misclassified point and the corresponding margin for the class. The soft-margin minimization process allows the ECOC SVM algorithm to differentiate between misclassified points that are close to being classified correctly and those that are far from being classified correctly. As a result, the ECOC SVM model is better than the *k*NN algorithm because it is less sensitive to noise in the data.

**FIGURE 10:**
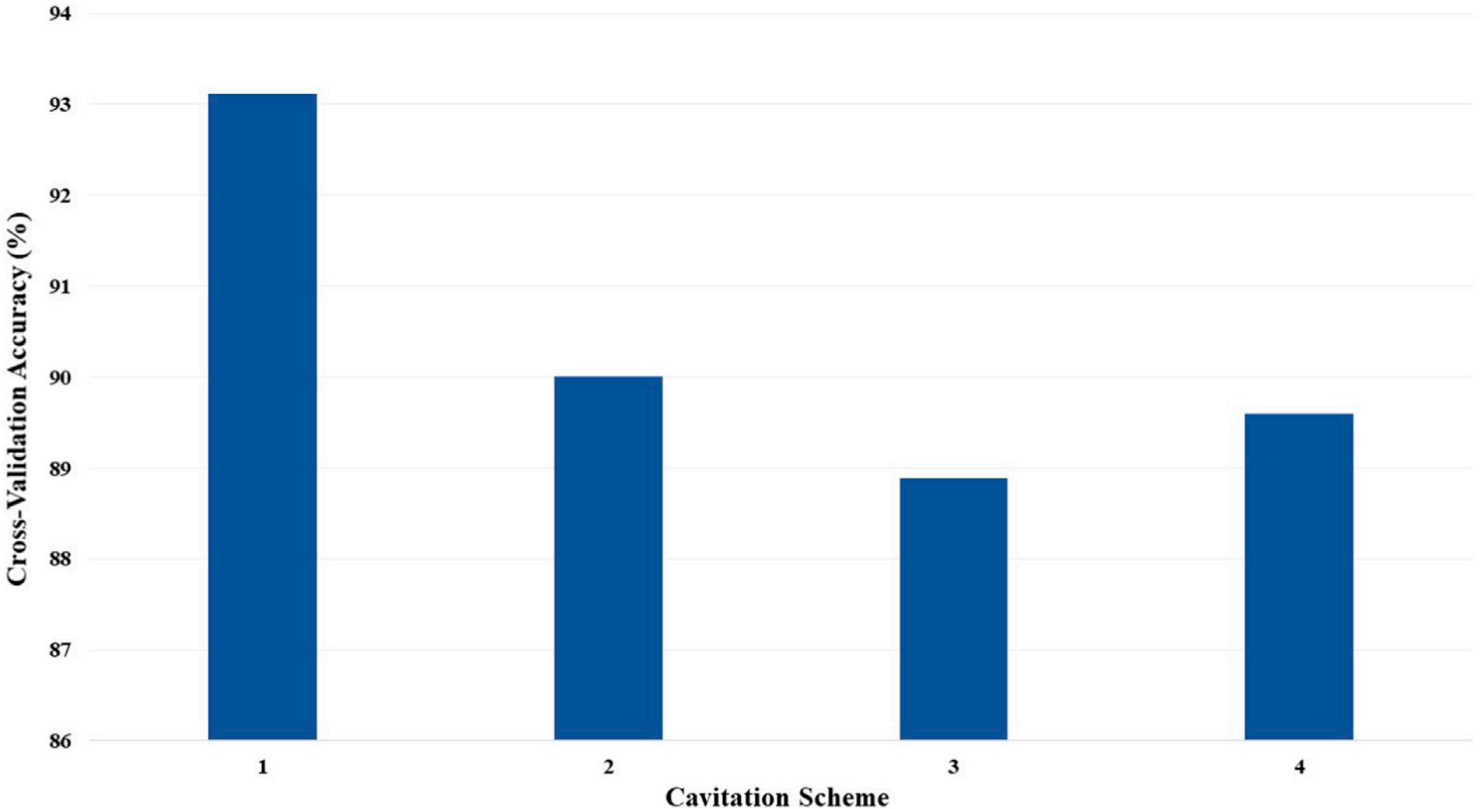
Cross-validation accuracy of the ECOC SVM models based on the cavitation scheme for cost *C* = 1.

Since the *C* is automatically processed for the ECOC SVM model, the performance is only compared across cavitation schemes. The cross-validation accuracies for the ECOC SVM models, by the cavitation scheme, are depicted in Figure 10.

**FIGURE 11:**
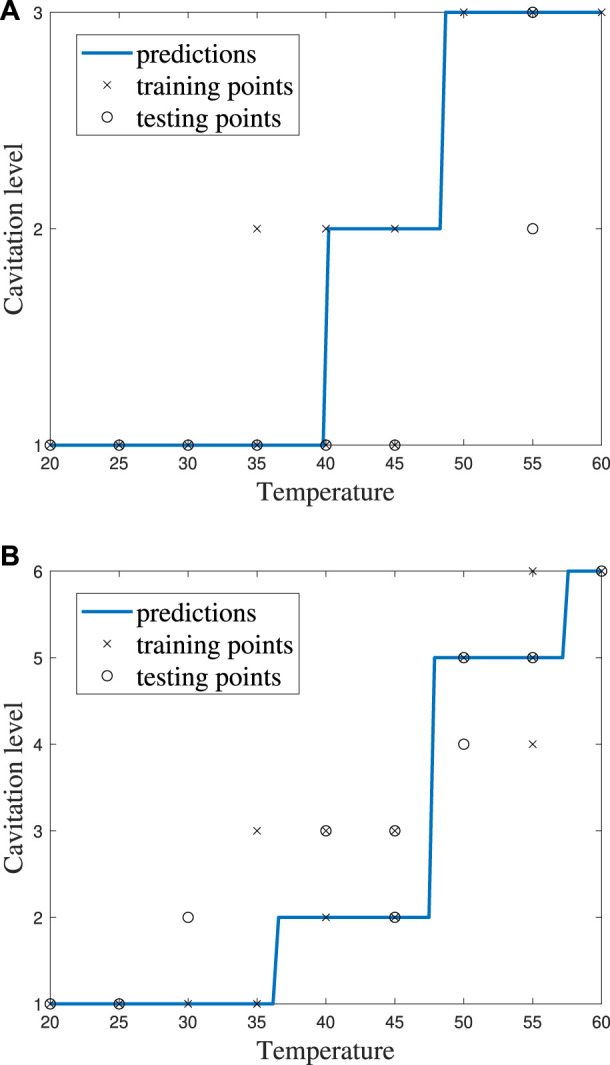
ECOC SVM model performance for cost *C* = 1 using **(A)** cavitation scheme 1 and **(B)** cavitation scheme 2.

**FIGURE 12:**
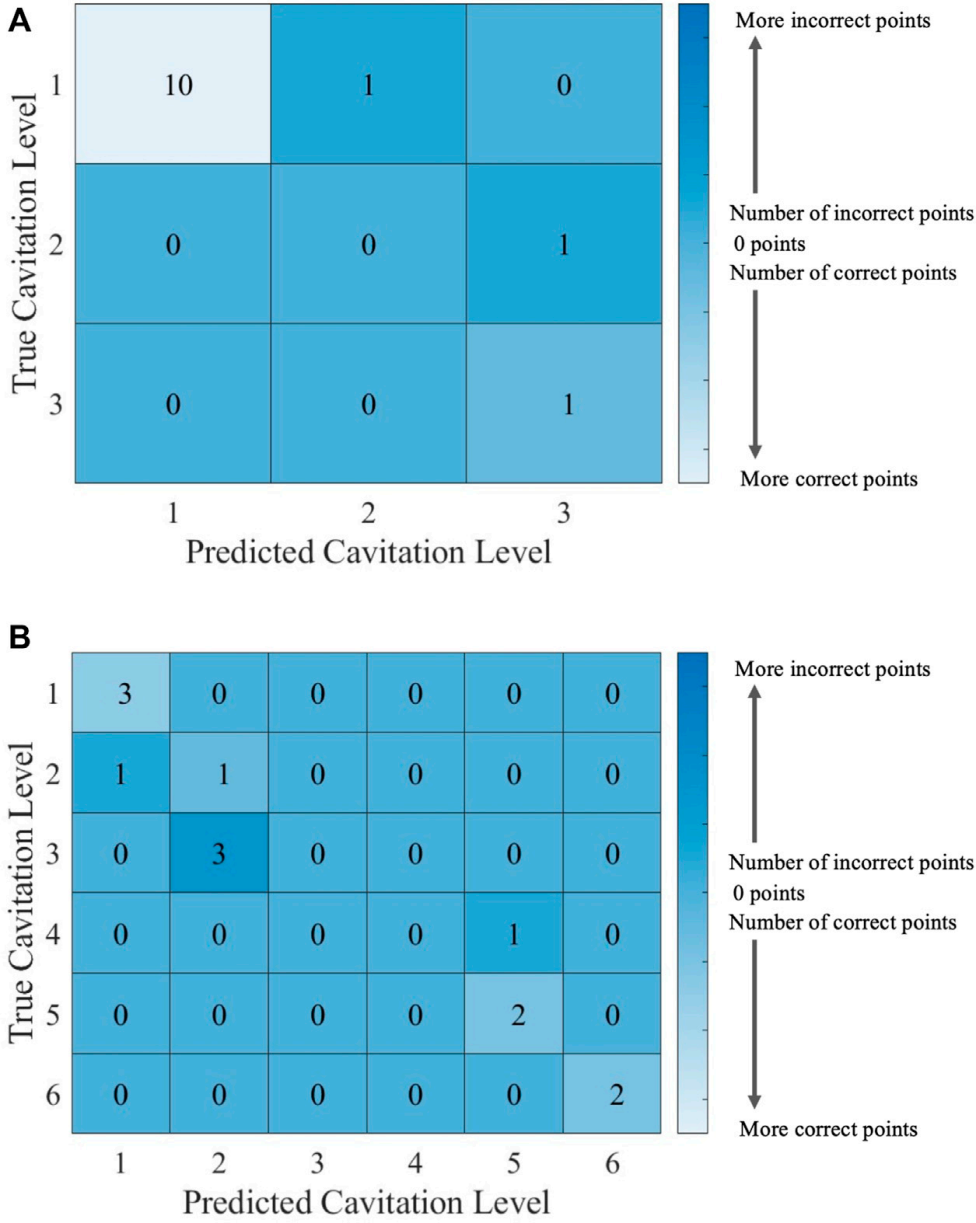
Confusion matrices for the ECOC SVM model with cost *C* = 1 using **(A)** cavitation scheme 1 and **(B)** cavitation scheme 2. The total number of rows and columns in the confusion matrix correspond to the number of cavitation levels defined by the cavitation schemes in Table 1.

Individual ECOC SVM model accuracy was depicted using a graphical representation (i.e., Figure 11), where a prediction line is used to illustrate when the model predicted the testing points. Confusion matrices for the predictions by the ECOC SVM model are also provided (i.e., Figure 12).

Supplementary Tables S5, S6, which can be found in Supplementary Materials, show the number of training points and testing points for the ECOC SVM models.

The cavitation scheme had a smaller overall effect on ECOC SVM performance when compared with the *k*NN’s performance. The simplest cavitation scheme (i.e., scheme 1) produced the highest accuracy of 93.11%. The second cavitation scheme performed only 4% worse than scheme 1, with an accuracy of 89.56%. Schemes 3 and 4 also had good accuracies of 88.89% and 89.56%, respectively. In all cases, the ECOC SVM model did at least 3% better than the best-performing *k*NN models in the data-driven schemes, but did up to 13% better than *k*NN for the distribution-driven cavitation schemes. The comparison of the two best-performing schemes (1 and 2) for the ECOC SVM is shown in Figure 11 and Figure 12. Specifically, the predictive performance for schemes 1 and 2 is shown in Figure 11, and the corresponding confusion matrices are shown in Figure 12.

The ECOC SVM model was able to generate much more accurate predictions of the cavitation level than the *k*NN models, regardless of the number of *k* neighbors chosen or the cavitation scheme used. Using a simpler cavitation scheme (i.e., scheme 1), both the *k*NN and ECOC SVM models produced a cross-validation accuracy above 85%, with the ECOC SVM obtaining almost 95% accuracy. Using the more complex cavitation scheme (i.e., scheme 4), ECOC SVM still outperforms the best *k*NN model (i.e., *k* = 3) by 11%. A comparison of the machine learning algorithms by the cavitation schemes can be seen in Table 3.

**TABLE 3:** Cross-validation accuracies for the four cavitation schemes as a function of all *k*NN and ECOC SVM machine learning models.

	Cross-validation accuracies			
	Scheme 1 (%)	Scheme 2 (%)	Scheme 3 (%)	Scheme 4 (%)
*k*NN with one neighbor	86.32	86.99	66.54	74.75
*k*NN with two neighbors	86.99	84.95	75.51	76.39
*k*NN with three neighbors	84.95	86.89	74.33	78.23
*k*NN with five neighbors	85.29	85.29	74.18	77.51
*k*NN with seven neighbors	86.32	86.32	70.55	69.43
ECOC SVM with *C* = 1	93.11	90.00	88.89	89.56

### 3.5 Confidence intervals from the bootstrapping method

The bootstrap mean accuracy, standard deviation, and 95% confidence intervals for each model and cavitation scheme are presented in Supplementary Materials (Sections 1.5 and 2.6). Overall, the bootstrapped mean accuracy was within 4% of the cross-validation accuracy, and several of the cross-validation accuracies fall into the bootstrapped confidence intervals. The bootstrapped mean accuracies for schemes 1, 3, and 4 were within 2% of the cross-validation accuracies. There was a larger difference (8.7%, on average) between the cross-validation and bootstrapped accuracies for cavitation scheme 2. The overall trends that cavitation scheme 1 produced the highest accuracy and that the ECOC SVM models had higher accuracy than the *k*NN models were consistent using both the cross-validation and bootstrap mean accuracy methods. Since cavitation schemes 1, 3, and 4 had more consistent results between both the cross-validation and bootstrap mean accuracy methods, this could be an indication that these schemes are more robust. Comparing cross-validation and bootstrapped accuracies could be a useful tool in determining the ideal cavitation scheme for a model. The benefits and limitations to each method for assessing accuracy are further evaluated in Section 4.

## 4 Discussion

The results from the present study indicate that machine learning algorithms like *k*NN and ECOC SVM are capable of accurately predicting fluid behavior (e.g., shock-induced cavitation) given a fluid condition like temperature. The *k*NN and ECOC SVM models were able to achieve 93.11% cross-validation accuracy even with a very small training set (*n* = 45) by machine learning standards (Shaikhina et al., 2015; Zhang and Ling, 2018). These findings support the use of machine learning methods to investigate cavitation as a mechanism of bTBI.

Cross-validation accuracies and bootstrapped mean estimates with confidence intervals were the two different methods applied to estimate the accuracy of the *k*NN and ECOC SVM models. The two approaches have various benefits and limitations. Cross-validation methods are simple to compute and require a low computational load (Koul et al., 2018), but the accuracy estimates obtained using cross-validation methods tend to have high variance depending on the specific train–test split (Koul et al., 2018). In contrast, bootstrapping methods are more complex and require a heavier computational load than cross-validation methods, but bootstrapping methods provide estimates that have lower variance (Wehrens et al., 2000). Therefore, bootstrapping methods may be more useful than cross-validation methods when the computational cost is not excessive. Overall, the two methods showed very good agreement for cavitation schemes 1, 3, and 4 (within 2%). Cavitation scheme 2 had an average percent difference of 8.7%, which may be attributed to the definitions of cavitation levels 5 and 6. The definitions of levels 1–4 were the same between cavitation schemes 2 and 3, but scheme 3 had much higher bootstrapping accuracies, which may indicate the variation is attributed to cavitation levels 5 and 6.

The results from the cross-validation accuracies and bootstrapped mean estimates suggest that both methods may be useful in determining the most accurate cavitation scheme. It is common to perform cross-validation for machine learning (Refaeilzadeh et al., 2009), and many current papers use hold-out cross validation, where the accuracy of the model is estimated based on the test accuracy of a model on a single test set (Koul et al., 2018). The cross-validation methodology used in this paper is an adapted hold-out technique described in Section 2.4.2, which took an average of 10 different models with different test sets and is thus even more reliable due to less dependence on the specific train–test split. Conversely, bootstrapping is much more time-consuming because it requires the training of each model type several times. In the bootstrapping methodology used in this paper, each model was trained 100 times, resulting in the overall training of 2,400 models. Each model was trained 100 times as per the recommendation by Bouckaert (2003) on how to choose between algorithms. Consequently, the mean and variance of the algorithms’ accuracies are estimated using 100 individual accuracies for each algorithm. The bootstrapping approach can be a less feasible option for a more costly training process, such as one that has a large amount of data or one that involves more complex models (i.e., neural networks that take longer to train) (Wehrens et al., 2000). For smaller datasets where the cost of training is comparatively low, the inclusion of bootstrapping methods can help establish confidence in model accuracy.

While the shock-induced cavitation data used for this study were not collected *in vivo* nor did it consider a biofluid such as cerebrospinal fluid in the chamber, the machine learning approach should be considered in the future to predict the influence of multiple biological parameters (e.g., temperature, salinity, pH, geometry, viscosity, and ion concentration) on biofluid behavior in blast environments. Although these machine learning models would inherently face some of the same non-parametric uncertainty as conventional models (e.g., the actual number or size of cavitation bubbles generated in the brain by shock exposure), the ability to validate each biological parameter’s effect in *ex vivo* experiments increases the likely biofidelity of the predicted biofluid behavior using *in vivo* models or computational simulations.

### 4.1 Comparison of *k*NN and SVM models

The machine learning models in this study only considered one feature (i.e., fluid temperature), which has a strong and positive correlation with the number of cavitation bubbles. The results consistently showed that the ECOC SVM outperforms the *k*NN when predicting the cavitation level based on fluid temperature. Using other data structures (e.g., non-linear data or data points that have multiple labels), the *k*NN may be preferred over the ECOC SVM because the ECOC SVM generally does not handle the other data structures well. Additionally, the *k*NN may also perform better than the ECOC SVM when the amount of training data relative to model features (i.e., data inputted into the machine learning model) is increased (Singh et al., 2016; Chahar and Kaur, 2020).

The ECOC SVM is generally considered simpler and more interpretable than the *k*NN but can only identify a smaller set of patterns when compared with the *k*NN (Singh et al., 2016; Chahar and Kaur, 2020). Thus, it follows that the ECOC SVM would perform well on the one feature data set because the pattern is simple. The ECOC SVM model also tends to perform better than the *k*NN when there are a large number of inputs relative to sample size (Yan and Yang, 2014; Gaye et al., 2021). As a result, it would be advantageous to include additional inputs like salinity, pH, geometry, viscosity, and ion concentration in an ECOC SVM model since this action would likely improve predictions of shock-induced cavitation behavior to investigate cavitation as a bTBI mechanism.

Both *k*NN and SVM are shallow and supervised learning methods. Shallow *versus* deep learning refers to the architecture of a model and the number of layers of representations these models contain (Zhong et al., 2019). Supervised *versus* unsupervised learning refers to whether or not the model is trained using labeled (by humans) training data or is allowed to define its own groups and clusters based on the model’s own detected features in unlabeled data (Rajoub, 2020). Both supervised and shallow learning methods are appropriate for labeled datasets with simple patterns and provide high accuracy when making predictions (Malach and Shalev-Shwartz, 2019; Robles Herrera et al., 2022). The pros and cons of a deep learning approach for future work with these types of data are discussed in Section 4.4.

In future, the machine learning model will be expanded by increasing the number of input parameters and datasets. The performance of the modified machine learning model, containing the expanded input and data, will be tested using other commonly used supervised learning algorithms (e.g., decision trees, random forest, and XGBoost) and compared with the results from the *k*NN and ECOC SVM. Decision trees are often used in healthcare research and can handle versatile data structures, which would be useful to consider in a version of the model that applies a wider set of inputs (Charbuty and Abdulazeez, 2021). Decision trees were not selected for the current work due to the model design (a single input and output with different numbers of levels). This is attributed to decision trees being biased toward more levels, which could have impaired the identification of the optimal cavitation scheme in this work. Random forest algorithms create a set of decision trees that are trained using different subsets of the training points. Random forest models can be more robust to overfitting than a single decision tree and can handle larger datasets. As a result, random forest models may be useful for a bTBI prediction model that considers a larger number of input parameters for cavitation and other medical data. The capability to handle larger datasets was not necessary in this work, particularly when weighted against the decreased interpretability of the results and the increased number of hyperparameters (e.g., number of trees, tree depth, and number of features) of a random forest algorithm when compared with the *k*NN or ECOC SVM model (Ao et al., 2019). XGBoost is a boosting method that trains multiple models sequentially to help improve each new decision tree. While the speed and versatility of XGBoost make it an appealing option for a model having multiple input parameters, the literature has shown that the XGBoost technique is a sub-optimal model for small datasets and was therefore not considered in this work (Azmi and Baliga, 2020).

### 4.2 Choice of *k*

The suggested *k*-value of 5, calculated using the square root of the training data, did not produce the highest cross-validation accuracy. Rather, the highest cross-validation accuracy (averaged over all cavitation schemes) was for a *k*-value of 3 (i.e., best *k*-value). There was a very small difference (less than 1%) between the average performance of the *k*NN model when using *k* nearest neighbor values of 2, 3, and 5. The *k*-values of 1 and 7 averaged 78% accuracy across all cavitation schemes, only 2%–2.5% less accurate than the other *k*-values. The results of the cross-validation highlight the importance of performing an analysis to determine the optimal choice of *k*. While the square root of the training data size is a good suggested starting point, the suggested *k*-value may not be the optimal *k* value for all data.

Although the ideal value of *k* may change in the data range (i.e., for each trial within a particular cavitation scheme), the best *k*-value was used in the analysis for simplicity (Garcia-Pedrajas et al., 2017). Thus, future work should consider the implementation of a data-driven *k* selection method where the optimal value of *k* is determined for each trial (Cheng et al., 2014). For instance, an adapted correlation matrix *k*NN could provide additional classification accuracy for the *k*NN model.

### 4.3 Choice of the cavitation scheme

A universal method of quantifying the level of cavitation using the bubble number does not exist. As such, this paper explored several different definitions of the cavitation level using the term “cavitation scheme” to analyze the predictive ability of the *k*NN and ECOC SVM models. The choice of the cavitation scheme impacted the predictive accuracy of *k*NN models strongly and moderately affected ECOC SVM models. Two approaches to defining cavitation levels were explored. The first approach is the “data-driven” cavitation scheme, which bases the distribution and definition of the cavitation level on the actual data from the shock tube experiments. The data-driven schemes were selected so that each possible cavitation level was reflective of at least one trial from the dataset. The second approach is the “distribution-driven” cavitation scheme, where a similar number of bubbles are selected in each level or “bin.” In the “distribution-driven” schemes, there may not be trials in the training data for a given bin (i.e., it is possible to have a bin without any data describing the number of cavitation bubbles).

Overall, the data-driven approach performed better than the distribution-driven approach. The simplest scheme (i.e., scheme 1) was defined using the data-driven approach and achieved the highest accuracy. This result is as expected since the more data points fit within each classified level/bin, the more training points the machine learning algorithms had to “learn” at each level. However, the other cavitation schemes using either the data-driven or distribution-driven approach were still able to achieve high accuracy when predicting the level of cavitation. This demonstrates the need for an ideal cavitation scheme definition that maximizes both predictive accuracy and result specificity. For example, if both cavitation schemes 1 and 2 yield an accuracy above 90%, it may be more beneficial to select the more detailed cavitation scheme (i.e., scheme 2 for this study).

### 4.4 Limitations and future directions

One limitation in the results presented in this paper is the small size of the dataset, which may result in the conclusions being a function of a random decision by the algorithms. However, despite the small size of the training dataset, the high accuracy obtained from the *k*NN and ECOC SVM algorithms supports continued efforts in applying machine learning to predict shock-induced cavitation. The present study demonstrates initial evidence that machine learning can be used to make predictions of cavitation behavior based on parameters related to blast injury and that these findings can be validated using a shock tube model.

This study only considered the *k*NN and ECOC SVM models since both have been considered in applications related to biology or cavitation. With more input parameters (e.g., ion concentration and viscosity), it is likely that ECOC SVM would continue to perform better than *k*NN. The current model is focused on predicting the cavitation bubble number (i.e., cavitation level). For other output parameters, like the strength of cavitation bubble collapse, it could also be useful to consider a different algorithm type (e.g., decision trees) that has been used in the literature (Wang et al., 2021). Additionally, as the data inputs increase in number and complexity, it could be useful to compare the performance of an unsupervised algorithm (i.e., *k*-means clustering) to the supervised ECOC SVM. As mentioned earlier, unsupervised machine learning describes algorithms that analyze unlabeled datasets without any human intervention (Rajoub, 2020). A key limitation to applying unsupervised learning to more complex data from clinical settings is interpretability. Since unsupervised algorithms may return categories that are not consistent with human-defined categories, the practical implications of the machine learning results can be difficult to interpret and pose challenges when evaluating their accuracy. Additionally, the *k*NN and ECOC SVM may be less prone to overfitting, when compared with unsupervised learning, since cross-validation methods can be used to help prevent overfitting in supervised learning (Koul et al., 2018). This is not easy in unsupervised learning due to the lack of labels associated with the data.

In this study, there was little value to applying unsupervised machine learning since a single, highly correlated input (i.e., fluid temperature) and output (i.e., cavitation level) were considered. Unsupervised learning has more potential benefit when there are complex patterns and interactions that a human might not be able to readily discern. In the context of bTBI, supervised learning would be beneficial to characterize singular, easily labeled outcomes (e.g., level of cavitation and presence or absence of coma). On the other hand, unsupervised learning methods could be more beneficial in detecting the underlying patterns in cavitation and intracranial pressure, which lead to moderate bTBI (Bröker et al., 2022).

The influence of chamber geometry on shock-induced cavitation was not assessed in this study, which is one limitation of this work. This limitation will be addressed in future work involving shock tube experiments using multiple chamber geometries to understand how the geometry will influence cavitation behavior. In this case, the machine learning algorithms would first be trained using the dimensions from multiple chamber geometries to see if cavitation can be predicted without the influence of geometry. If the prediction ability of the machine learning algorithms using the different geometries is poor, then the geometry would be added as a model input feature which will be experimentally validated.

While the *k*NN and ECOC SVM models presented in this study predicted shock-induced cavitation behavior based on the fluid property temperature, there may be an application where the machine learning model could predict the level of shock-induced cavitation that could lead to a bTBI. In this case, cavitation above a certain level would result in a model prediction of bTBI, while cavitation below that level would result in a prediction of no bTBI. To achieve something like this, machine learning could be coupled with experiments or simulations. The model would be given the blast and fluid conditions (overpressure, temperature, etc.) as inputs and would output whether a bTBI would be incurred or not, based on the predicted level of cavitation. This machine learning model may also be able to make predictive conclusions about the severity of the bTBI using the predicted level of cavitation. In the same way that Fadaei Kermani et al. (2018) assigned a cavitation index to a level of damage severity, the cavitation levels defined in this study could be assigned to levels of bTBI severity. For example, using cavitation scheme 1, level 1 could correlate to mild bTBI, level 2 is moderate, and level 3 is severe. These levels could be validated using real patient data, where the diagnosed bTBI severity is the output, and consider different input conditions such as age, presence of lesions in the brain, diagnostic scores, and magnitude of the overpressure (i.e., shock wave). Such a model could provide high clinical value due to the current ethical and practical challenges in quantifying brain injury severity and predicting injury outcomes. However, this model would require detailed knowledge of the injury conditions, and clinicians might be hesitant to discharge patients or recommend care plans based on the output from the algorithm. That being said, existing studies have already demonstrated the use of machine learning algorithms to predict patient outcomes or brain injury severity (Vergara et al., 2017; 2018; Hale et al., 2018). Despite the logistical obstacles posed by clinical implementation of machine learning algorithms, the low-risk nature of a potentially transformative reward supports investigation into this avenue. While ultimate medical recommendations should incorporate medical expertise and a variety of tests, machine learning is a potential option for clinicians to use patient data to help inform diagnostics and treatment plans. In the case of a heterogeneous condition like blast injury, models that can help accurately classify injury severity and predict outcomes can be a useful tool in the overall kit used to improve patient care.

This study further demonstrates the ability of machine learning to predict cavitation behavior in a fluid with good accuracy, which has implications for blast-injury models.

The machine learning algorithms presented in this study have the potential to quantify injury severity based on a mechanistic metric. If future machine learning models are adapted to predict injury severity based on the predicted cavitation level, then this would provide substantial support to the theory that cavitation is the main mechanism driving bTBI.

Consequently, the results of the present study emphasize the novel benefit that machine learning can offer for understanding the mechanisms of bTBI and predicting outcomes following this injury.

#### 4.4.1 Permission to reuse and copyright

Figures, tables, and images will be published under a Creative Commons CC-BY license, and permission must be obtained for use of copyrighted material from other sources (including re-published/adapted/modified/partial figures and images from the internet). It is the responsibility of the authors to acquire the licenses, follow any citation instructions requested by third-party rights holders, and cover any supplementary charges.

## Data Availability

The raw data supporting the conclusion of this article will be made available by the authors, without undue reservation.
