# Supplementary material for: Predicting shock-induced cavitation using machine learning: implications for blast-injury models
Source: Front Bioeng Biotechnol. 2024 Feb 5;12:1268314. doi: 10.3389/fbioe.2024.1268314 (PMC10877722; doi:10.3389/fbioe.2024.1268314)
Supplement: Supplementary file 1 [file DataSheet1.pdf]

frontiers

Supplementary Material

1 SUPPLEMENTARY TABLES

The supplementary tables show the number of training and testing points for each temperature and cavitation level, when considering selected k-values (applied in the k-Nearest Neighbors (kNN) model) and the adapted Support Vector Machine model, using Error-Correcting Output Codes (ECOC SVM). Tables containing the bootstrapping results for both the kNN and ECOC SVM models are also provided.

1.1 Training and Testing Points using the kNN Model and Cavitation Scheme 1

Table S1. Number of training and testing points, as a function of temperature and cavitation level, for the kNN Model using cavitation scheme 1. The $k = 2$ (best performing value of k) data from Figures S2a and S7a are used in the table.

		**Number of Training and Testing Points for Scheme 1 with $k = 2$**								
		20°C	25°C	30°C	35°C	40°C	45°C	50°C	55°C	60°C
Cavitation Level 1	Training Point(s)	3	5	4	3	3	1	0	0	0
	Testing Point(s)	2	0	1	1	0	1	0	0	0
Cavitation Level 2	Training Point(s)	0	0	0	0	1	2	0	0	0
	Testing Point(s)	0	0	0	1	1	1	0	1	0
Cavitation Level 3	Training Point(s)	0	0	0	0	0	0	4	2	4
	Testing Point(s)	0	0	0	0	0	0	1	2	1

Table S2. Number of training and testing points, as a function of temperature and cavitation level, for the kNN Model using cavitation scheme 1. The $k = 3$ (worst performing value of k) data from Figures S3a and S8a are used in the table.

		**Number of Training and Testing Points for Scheme 1 with $k = 3$**								
		20°C	25°C	30°C	35°C	40°C	45°C	50°C	55°C	60°C
Cavitation Level 1	Training Point(s)	4	3	3	3	1	2	0	0	0
	Testing Point(s)	1	2	2	1	2	0	0	0	0
Cavitation Level 2	Training Point(s)	0	0	0	1	1	3	0	0	0
	Testing Point(s)	0	0	0	0	1	0	0	1	0
Cavitation Level 3	Training Point(s)	0	0	0	0	0	0	5	4	2
	Testing Point(s)	0	0	0	0	0	0	0	0	3

Table S3. Number of training and testing points, as a function of temperature and cavitation level, for the kNN Model using cavitation scheme 1. The $k = 5$ data from Figures S4a and S9a are used in the table.

		**Number of Training and Testing Points for Scheme 1 with $k = 5$**								
		20°C	25°C	30°C	35°C	40°C	45°C	50°C	55°C	60°C
Cavitation Level 1	Training Point(s)	4	1	5	3	2	1	0	0	0
	Testing Point(s)	1	4	0	1	1	1	0	0	0
Cavitation Level 2	Training Point(s)	0	0	0	0	2	3	0	1	0
	Testing Point(s)	0	0	0	1	0	0	0	0	0
Cavitation Level 3	Training Point(s)	0	0	0	0	0	0	3	3	4
	Testing Point(s)	0	0	0	0	0	0	2	1	1

1.2 Training and Testing Points using the kNN Model and Cavitation Scheme 2

Table S4. Number of training and testing points, as a function of temperature and cavitation level, for the kNN Model using cavitation scheme 2. The $k = 5$ data from Figures S4b and S9b are used in the table.

		**Number of Training and Testing Points for Scheme 2 with $k = 5$**								
		20°C	25°C	30°C	35°C	40°C	45°C	50°C	55°C	60°C
Cavitation Level 1	Training Point(s)	4	4	2	2	0	0	0	0	0
	Testing Point(s)	1	1	2	2	0	0	0	0	0
Cavitation Level 2	Training Point(s)	0	0	1	0	3	1	0	0	0
	Testing Point(s)	0	0	0	0	0	1	0	0	0
Cavitation Level 3	Training Point(s)	0	0	0	0	1	2	0	0	0
	Testing Point(s)	0	0	0	1	1	1	0	0	0
Cavitation Level 4	Training Point(s)	0	0	0	0	0	0	1	1	0
	Testing Point(s)	0	0	0	0	0	0	0	0	0
Cavitation Level 5	Training Point(s)	0	0	0	0	0	0	3	2	0
	Testing Point(s)	0	0	0	0	0	0	1	1	0
Cavitation Level 6	Training Point(s)	0	0	0	0	0	0	0	0	5
	Testing Point(s)	0	0	0	0	0	0	0	1	0

1.3 Training and Testing Points using the ECOC SVM Model and Cavitation Scheme 1

Table S5. Number of training and testing points, as a function of temperature and cavitation level, for the ECOC SVM Model with cost $C = 1$ using cavitation scheme 1. The data from Figure S12a and S13a are used in the table.

		Number of Training and Testing Points for Scheme 1 with $C = 1$								
		20°C	25°C	30°C	35°C	40°C	45°C	50°C	55°C	60°C
Cavitation Level 1	Training Point(s)	2	4	3	2	1	1	0	0	0
	Testing Point(s)	3	1	2	2	2	1	0	0	0
Cavitation Level 2	Training Point(s)	0	0	0	1	2	3	0	0	0
	Testing Point(s)	0	0	0	0	0	0	0	1	0
Cavitation Level 3	Training Point(s)	0	0	0	0	0	0	5	3	5
	Testing Point(s)	0	0	0	0	0	0	0	1	0

1.4 Training and Testing Points using the ECOC SVM Model and Cavitation Scheme 2

Table S6. Number of training and testing points, as a function of temperature and cavitation level, for the ECOC SVM Model with cost $C = 1$ using cavitation scheme 2. The data from Figure S12b and S13b are used in the table.

		Number of Training and Testing Points for Scheme 2 with $C = 1$								
		20°C	25°C	30°C	35°C	40°C	45°C	50°C	55°C	60°C
Cavitation Level 1	Training Point(s)	3	4	4	4	0	0	0	0	0
	Testing Point(s)	2	1	0	0	0	0	0	0	0
Cavitation Level 2	Training Point(s)	0	0	0	0	3	1	0	0	0
	Testing Point(s)	0	0	1	0	0	1	0	0	0
Cavitation Level 3	Training Point(s)	0	0	0	1	1	1	0	0	0
	Testing Point(s)	0	0	0	0	1	2	0	0	0
Cavitation Level 4	Training Point(s)	0	0	0	0	0	0	0	1	0
	Testing Point(s)	0	0	0	0	0	0	1	0	0
Cavitation Level 5	Training Point(s)	0	0	0	0	0	0	3	2	0
	Testing Point(s)	0	0	0	0	0	0	1	1	0
Cavitation Level 6	Training Point(s)	0	0	0	0	0	0	0	1	3
	Testing Point(s)	0	0	0	0	0	0	0	0	2

1.5 Bootstrapping Results for the kNN and ECOC SVM Models

Table S7. Bootstrap estimates of mean accuracies, standard deviation, and confidence intervals for all models for cavitation scheme 1.

**Cavitation Scheme 1**			
Model	Bootstrap Estimate of Mean Accuracy	Standard Deviation	95% Confidence Interval
kNN with 1 neighbor	0.8656	0.0098	(0.8595, 0.8717)
kNN with 2 neighbors	0.8430	0.0110	(0.8361, 0.8498)
kNN with 3 neighbors	0.8707	0.0070	(0.8663, 0.8750)
kNN with 5 neighbors	0.8652	0.0104	(0.8588, 0.8716)
kNN with 7 neighbors	0.8555	0.0103	(0.8492, 0.8619)
ECOC SVM with $C = 1$	0.9478	0.0075	(0.9431, 0.9524)

Table S8. Bootstrap estimates of mean accuracies, standard deviation, and confidence intervals for all models for cavitation scheme 2.

**Cavitation Scheme 2**			
Model	Bootstrap Estimate of Mean Accuracy	Standard Deviation	95% Confidence Interval
kNN with 1 neighbor	0.7584	0.0165	(0.7482, 0.7687)
kNN with 2 neighbors	0.7821	0.0149	(0.7728, 0.7913)
kNN with 3 neighbors	0.7923	0.0105	(0.7859, 0.7988)
kNN with 5 neighbors	0.7875	0.0135	(0.7792, 0.7958)
kNN with 7 neighbors	0.7636	0.0124	(0.7559, 0.7713)
ECOC SVM with $C = 1$	0.9144	0.0060	(0.9107, 0.9182)

Table S9. Bootstrap estimates of mean accuracies, standard deviation, and confidence intervals for all models for cavitation scheme 3.

**Cavitation Scheme 3**			
Model	Bootstrap Estimate of Mean Accuracy	Standard Deviation	95% Confidence Interval
kNN with 1 neighbor	0.6768	0.0219	(0.6633, 0.6904)
kNN with 2 neighbors	0.7309	0.0195	(0.7188, 0.7430)
kNN with 3 neighbors	0.7556	0.0167	(0.7452, 0.7660)
kNN with 5 neighbors	0.7287	0.0156	(0.7190, 0.7384)
kNN with 7 neighbors	0.6921	0.0125	(0.6844, 0.6999)
ECOC SVM with $C = 1$	0.8827	0.0068	(0.8785, 0.8869)

Table S10. Bootstrap estimates of mean accuracies, standard deviation, and confidence intervals for all models for cavitation scheme 4.

**Cavitation Scheme 4**			
Model	Bootstrap Estimate of Mean Accuracy	Standard Deviation	95% Confidence Interval
kNN with 1 neighbor	0.7544	0.0192	(0.7425, 0.7663)
kNN with 2 neighbors	0.7736	0.0127	(0.7657, 07814)
kNN with 3 neighbors	0.7778	0.0117	(0.7706, 0.7850)
kNN with 5 neighbors	0.7604	0.0117	(0.7532, 0.7677)
kNN with 7 neighbors	0.7051	0.0120	(0.6977, 0.7126)
ECOC SVM with $C = 1$	0.8987	0.0069	(0.8944, 0.9029)

2 SUPPLEMENTARY FIGURES

The supplementary figures show the performances and corresponding confusion matrices for the k-Nearest Neighbors (kNN) models and an adapted Support Vector Machine model, using Error-Correcting Output Codes (ECOC SVM). The results from ten (10) kNN and ECOC SVM models, each using a different 70% training and 30% testing data, were averaged to obtain the cross-validation accuracies. The maximum and minimum cross-validation accuracies across all k values for each cavitation scheme are shown in a bar chart in Figure S11. Bootstrapping results for both the kNN and ECOC SVM models are shown in Figure S14 as a box-and-whisker plot.

2.1 kNN Model Performance

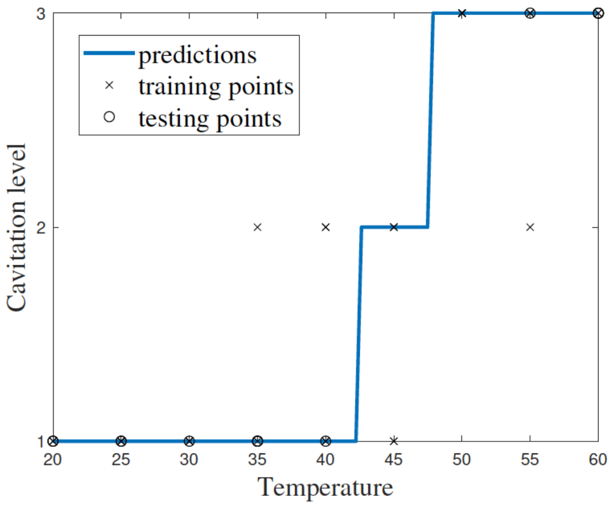

(S1a) Cavitation Scheme 1

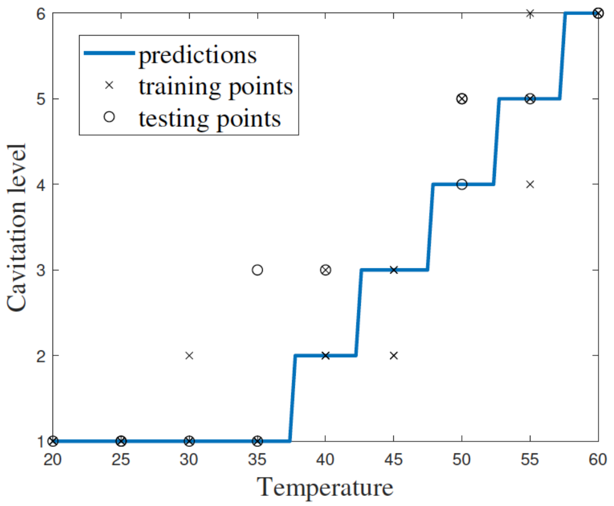

(S1b) Cavitation Scheme 2

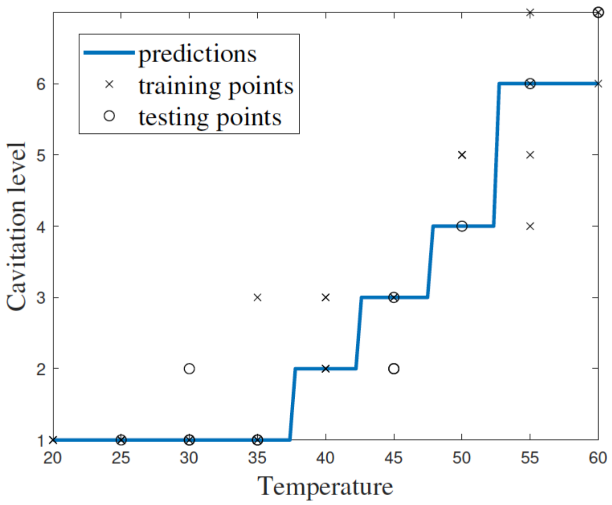

(S1c) Cavitation Scheme 3

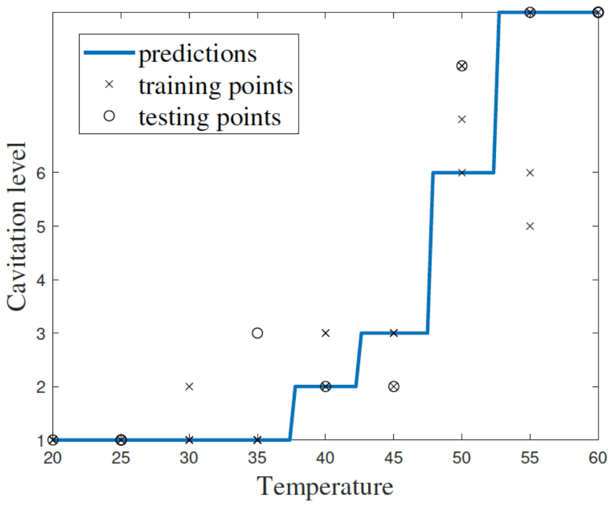

(S1d) Cavitation Scheme 4

Figure S1. kNN Model Performance for $k = 1$ using **(S1a)** cavitation scheme 1, **(S1b)** cavitation scheme 2, **(S1c)** cavitation scheme 3, and **(S1d)** cavitation scheme 4.

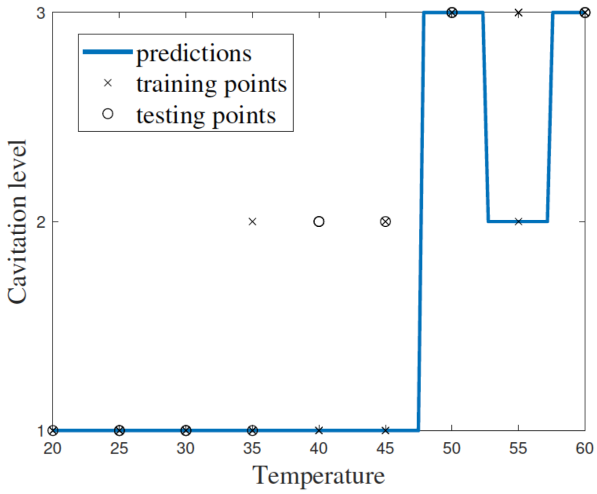

(S2a) Cavitation Scheme 1

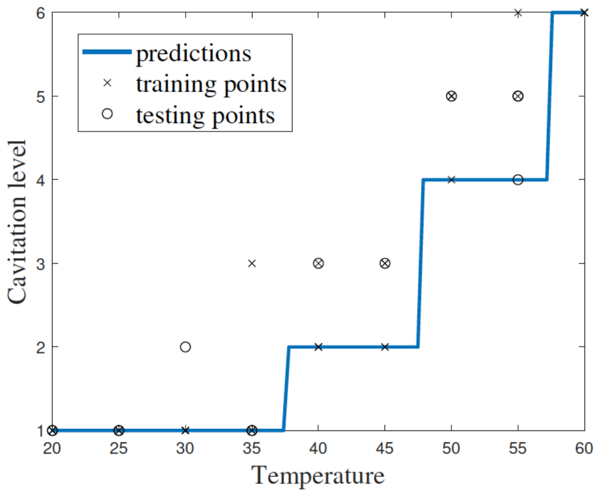

(S2b) Cavitation Scheme 2

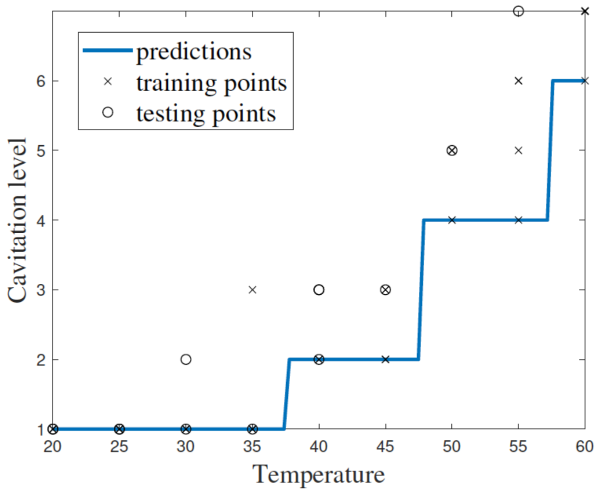

(S2c) Cavitation Scheme 3

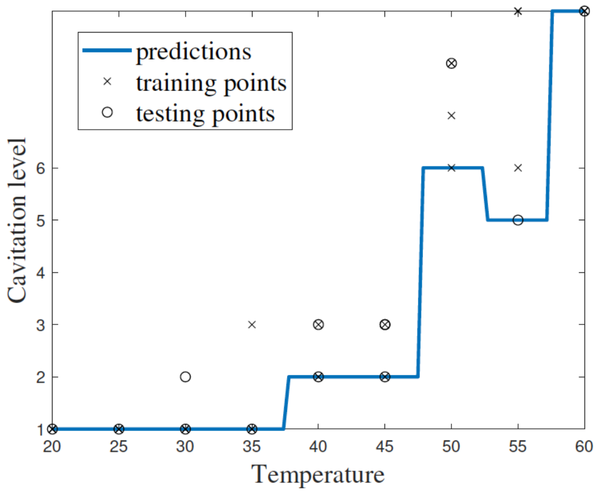

(S2d) Cavitation Scheme 4

Figure S2. kNN Model Performance for $k = 2$ using **(S2a)** cavitation scheme 1, **(S2b)** cavitation scheme 2, **(S2c)** cavitation scheme 3, and **(S2d)** cavitation scheme 4.

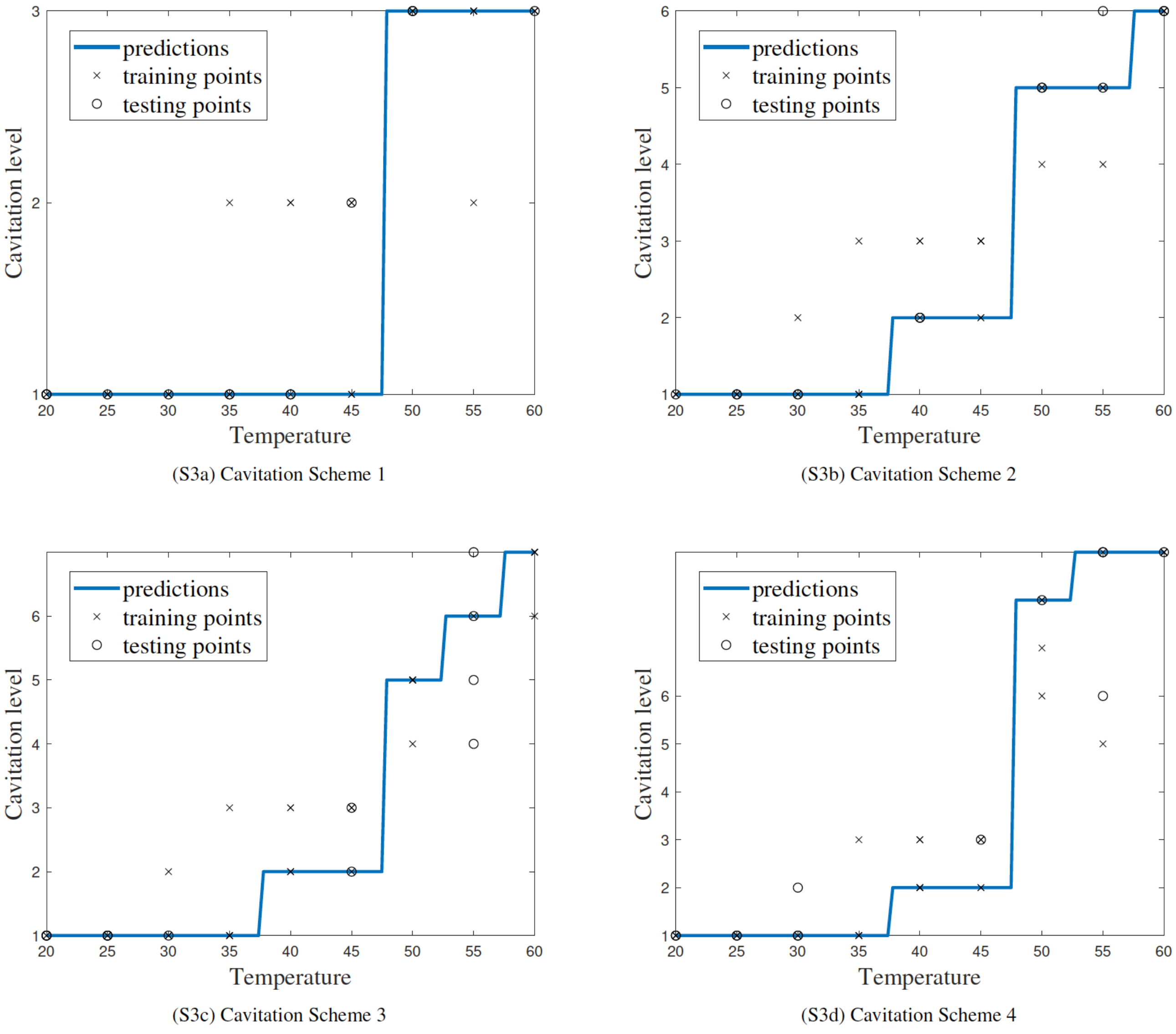

(S3a) Cavitation Scheme 1

(S3b) Cavitation Scheme 2

(S3c) Cavitation Scheme 3

(S3d) Cavitation Scheme 4

Figure S3. kNN Model Performance for $k = 3$ using **(S3a)** cavitation scheme 1, **(S3b)** cavitation scheme 2, **(S3c)** cavitation scheme 3, and **(S3d)** cavitation scheme 4.

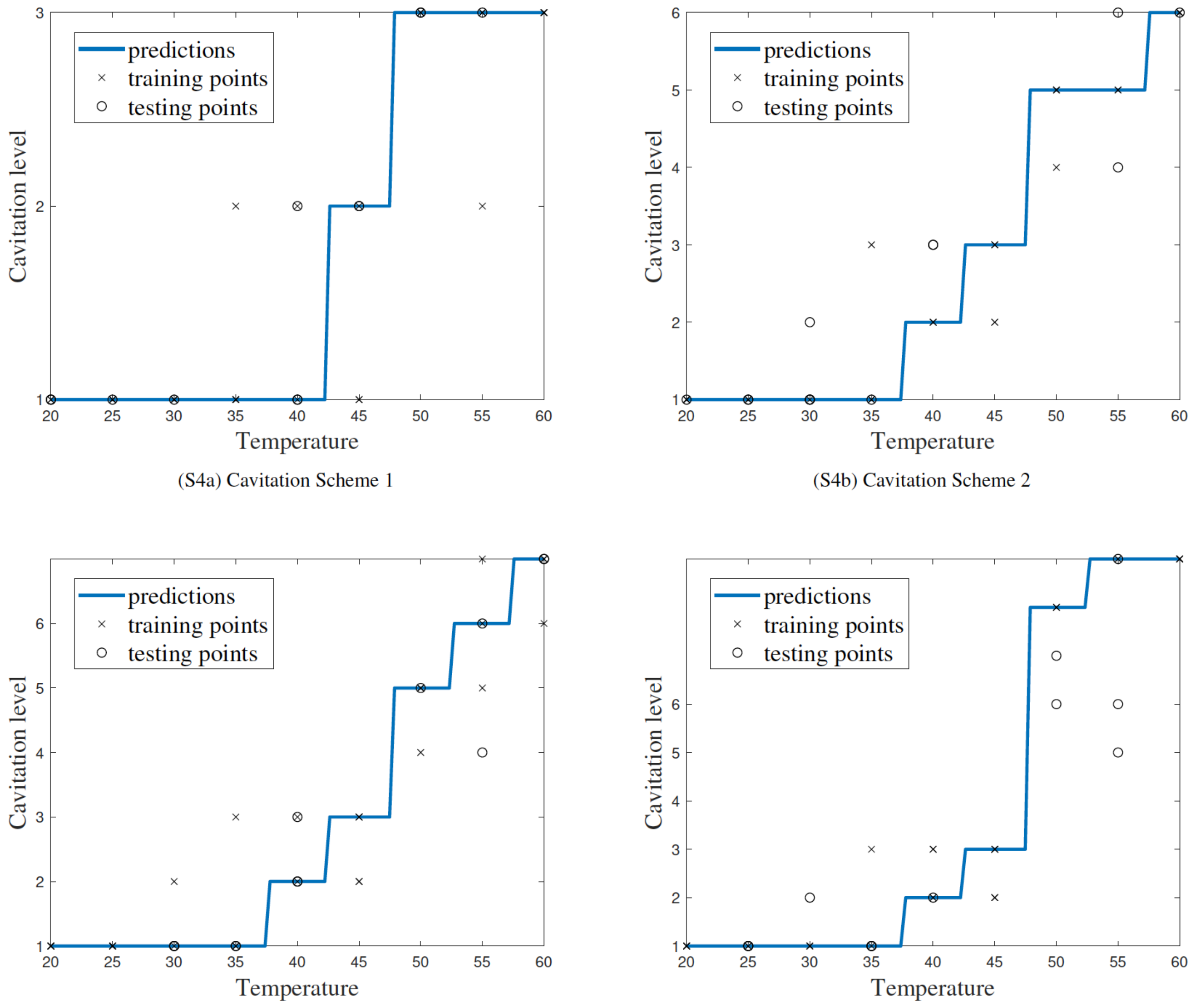

(S4a) Cavitation Scheme 1

(S4b) Cavitation Scheme 2

(S4c) Cavitation Scheme 3

(S4d) Cavitation Scheme 4

Figure S4. kNN Model Performance for $k = 5$ using **(S4a)** cavitation scheme 1, **(S4b)** cavitation scheme 2, **(S4c)** cavitation scheme 3, and **(S4d)** cavitation scheme 4.

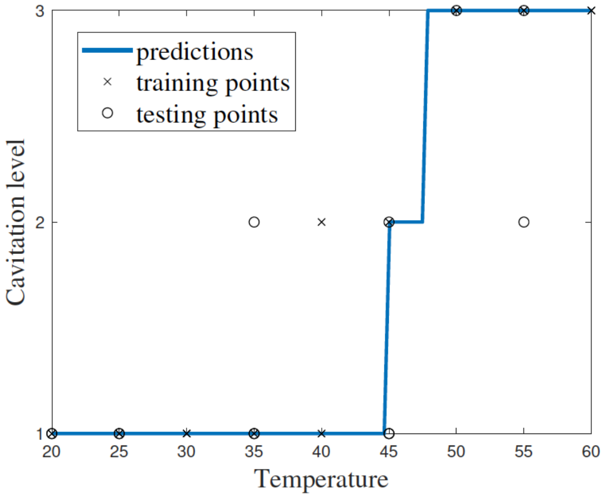

(S5a) Cavitation Scheme 1

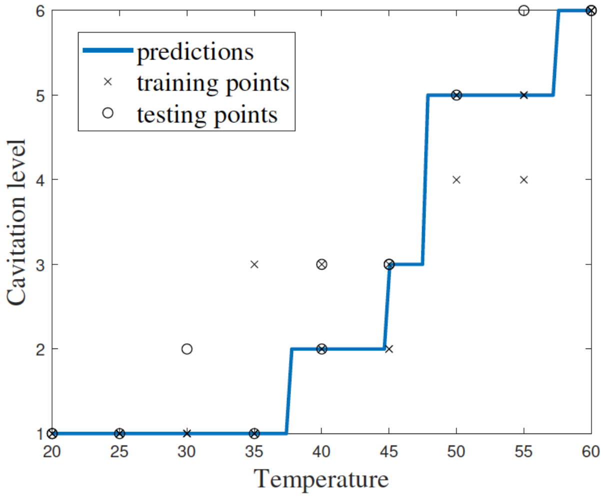

(S5b) Cavitation Scheme 2

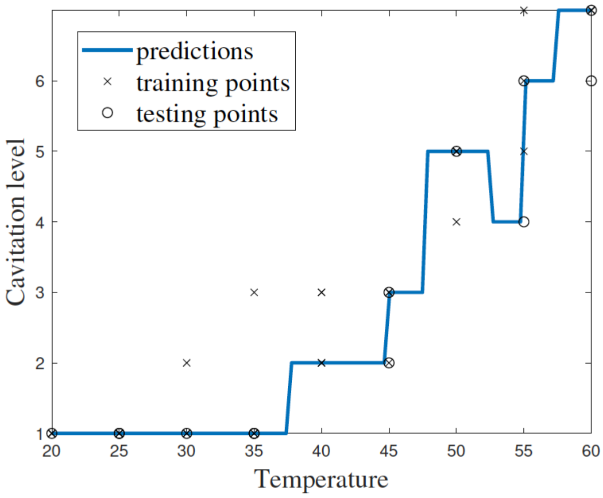

(S5c) Cavitation Scheme 3

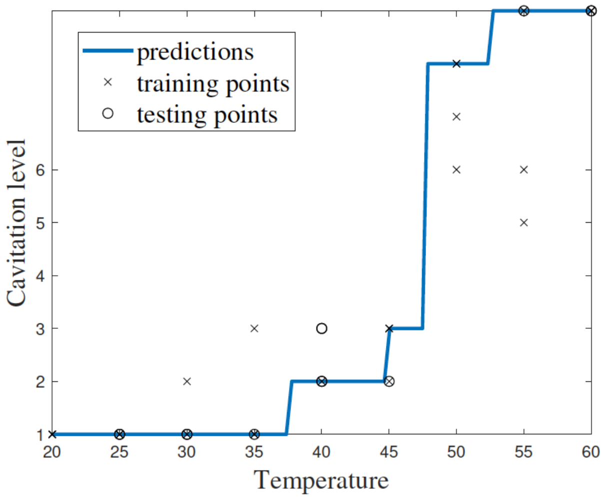

(S5d) Cavitation Scheme 4

Figure S5. kNN Model Performance for $k = 7$ using **(S5a)** cavitation scheme 1, **(S5b)** cavitation scheme 2, **(S5c)** cavitation scheme 3, and **(S5d)** cavitation scheme 4.

2.2 Confusion Matrices for the kNN Model

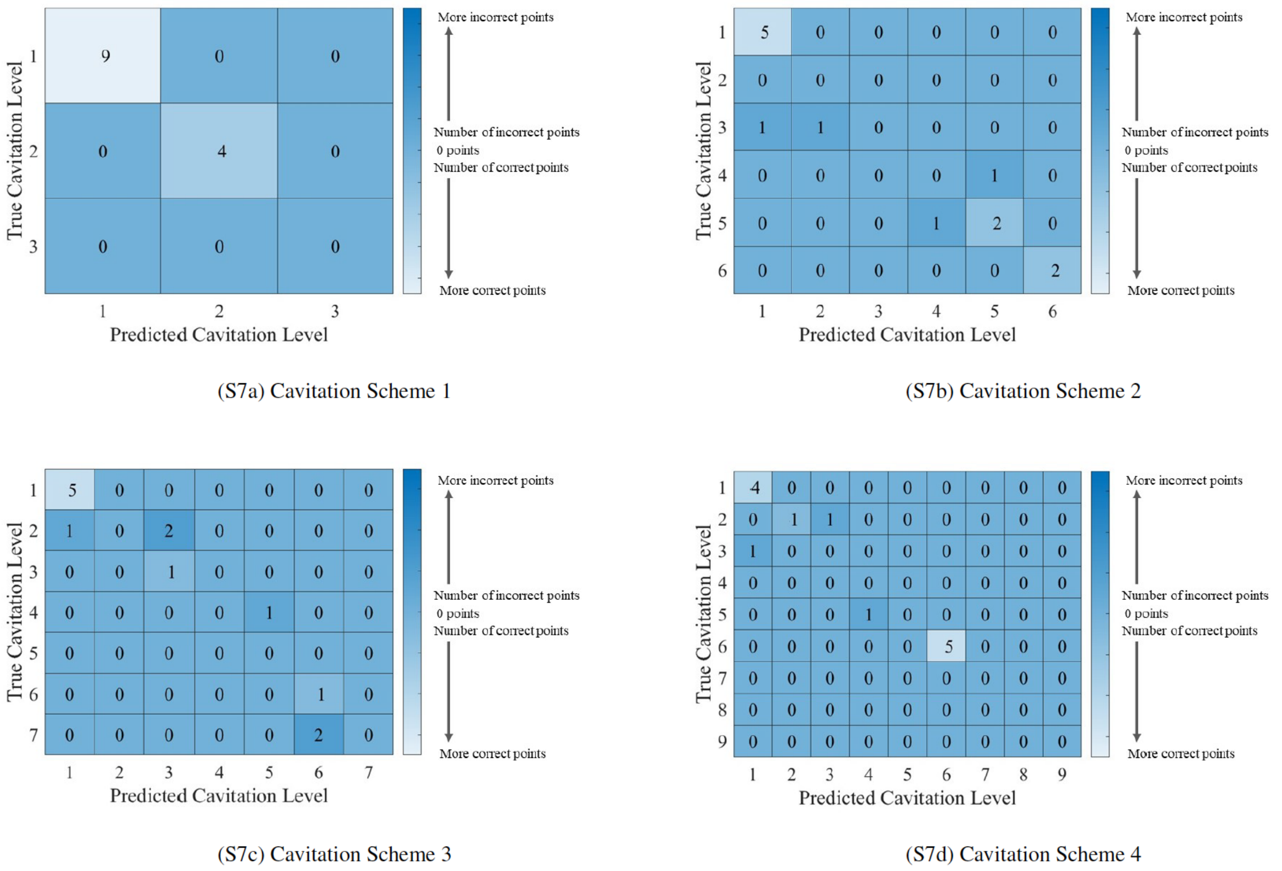

Figure S6. Confusion Matrices for the kNN model with $k = 1$ using **(S6a)** cavitation scheme 1, **(S6b)** cavitation scheme 2, **(S6c)** cavitation scheme 3, and **(S6d)** cavitation scheme 4.

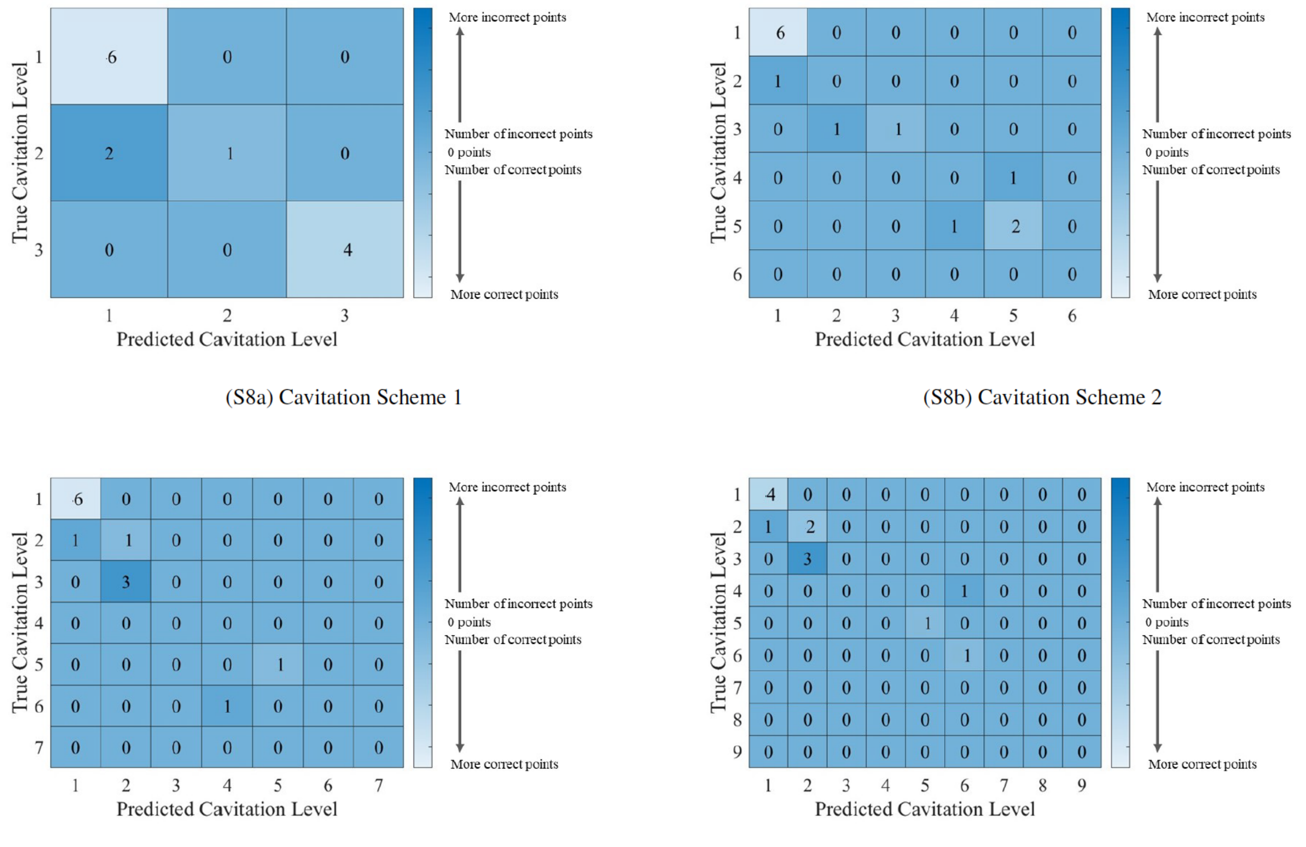

(S8a) Cavitation Scheme 1

(S8b) Cavitation Scheme 2

(S8c) Cavitation Scheme 3

(S8d) Cavitation Scheme 4

Figure S7. Confusion Matrices for the kNN model with $k = 2$ using **(S7a)** cavitation scheme 1, **(S7b)** cavitation scheme 2, **(S7c)** cavitation scheme 3, and **(S7d)** cavitation scheme 4.

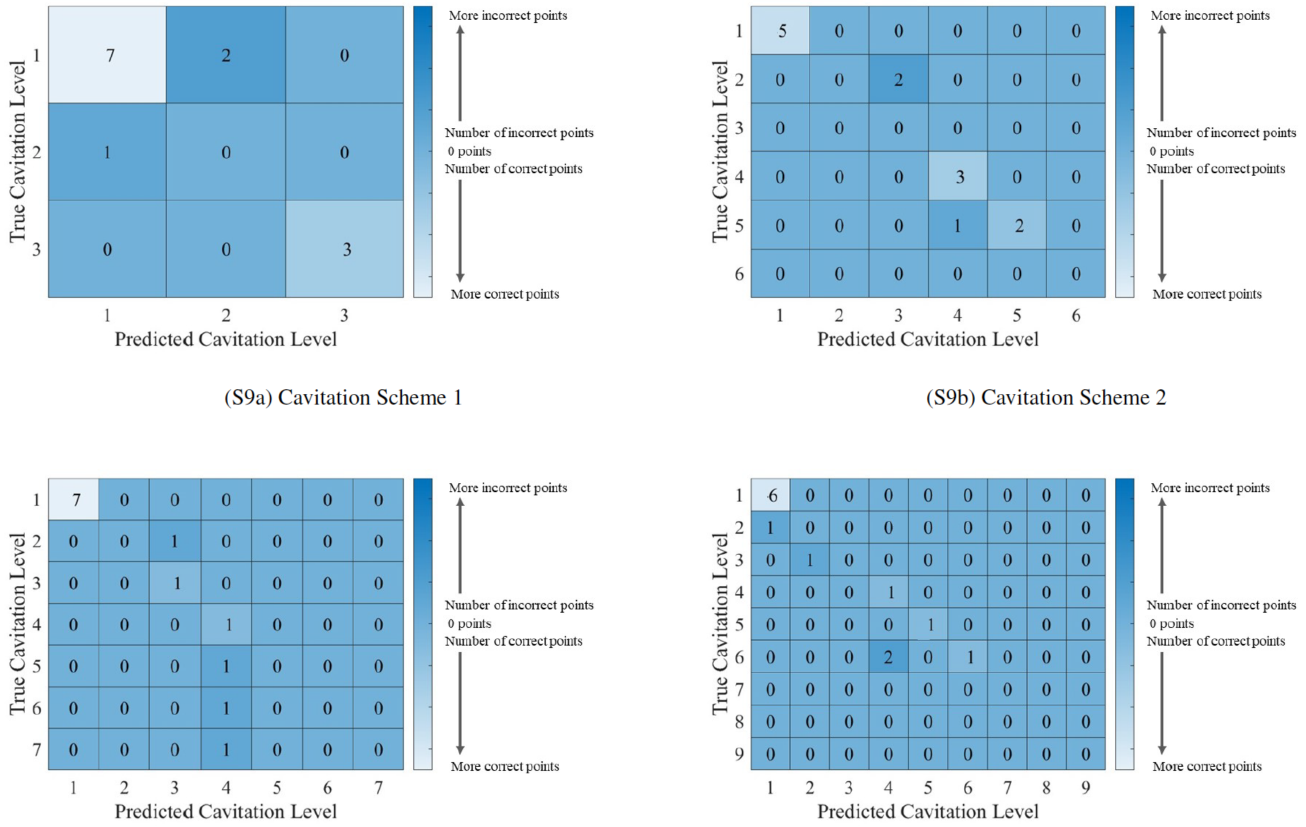

(S9a) Cavitation Scheme 1

(S9b) Cavitation Scheme 2

(S9c) Cavitation Scheme 3

(S9d) Cavitation Scheme 4

Figure S8. Confusion Matrices for the kNN model with $k = 3$ using **(S8a)** cavitation scheme 1, **(S8b)** cavitation scheme 2, **(S8c)** cavitation scheme 3, and **(S8d)** cavitation scheme 4.

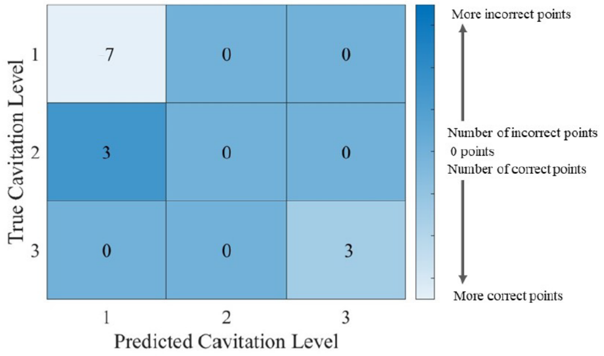

(S10a) Cavitation Scheme 1

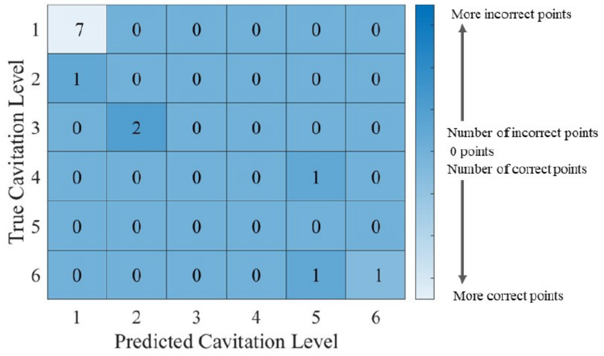

(S10b) Cavitation Scheme 2

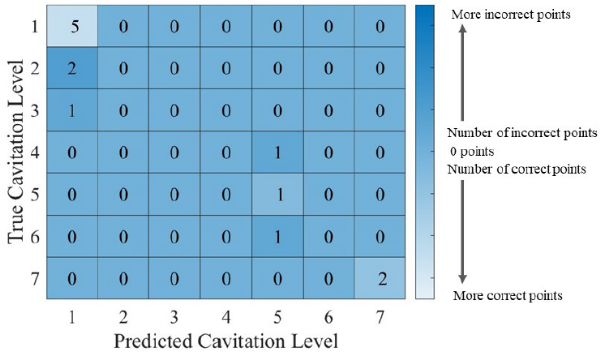

(S10c) Cavitation Scheme 3

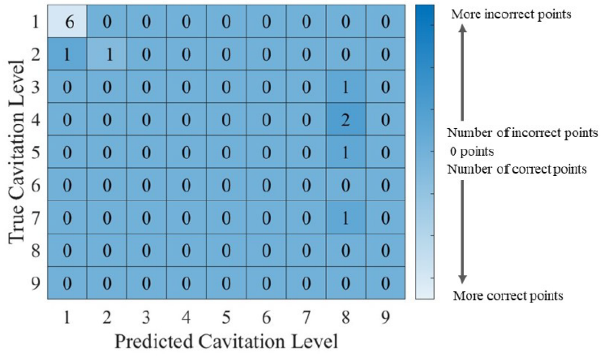

(S10d) Cavitation Scheme 4

Figure S9. Confusion Matrices for the kNN model with $k = 5$ using **(S9a)** cavitation scheme 1, **(S9b)** cavitation scheme 2, **(S9c)** cavitation scheme 3, and **(S9d)** cavitation scheme 4.

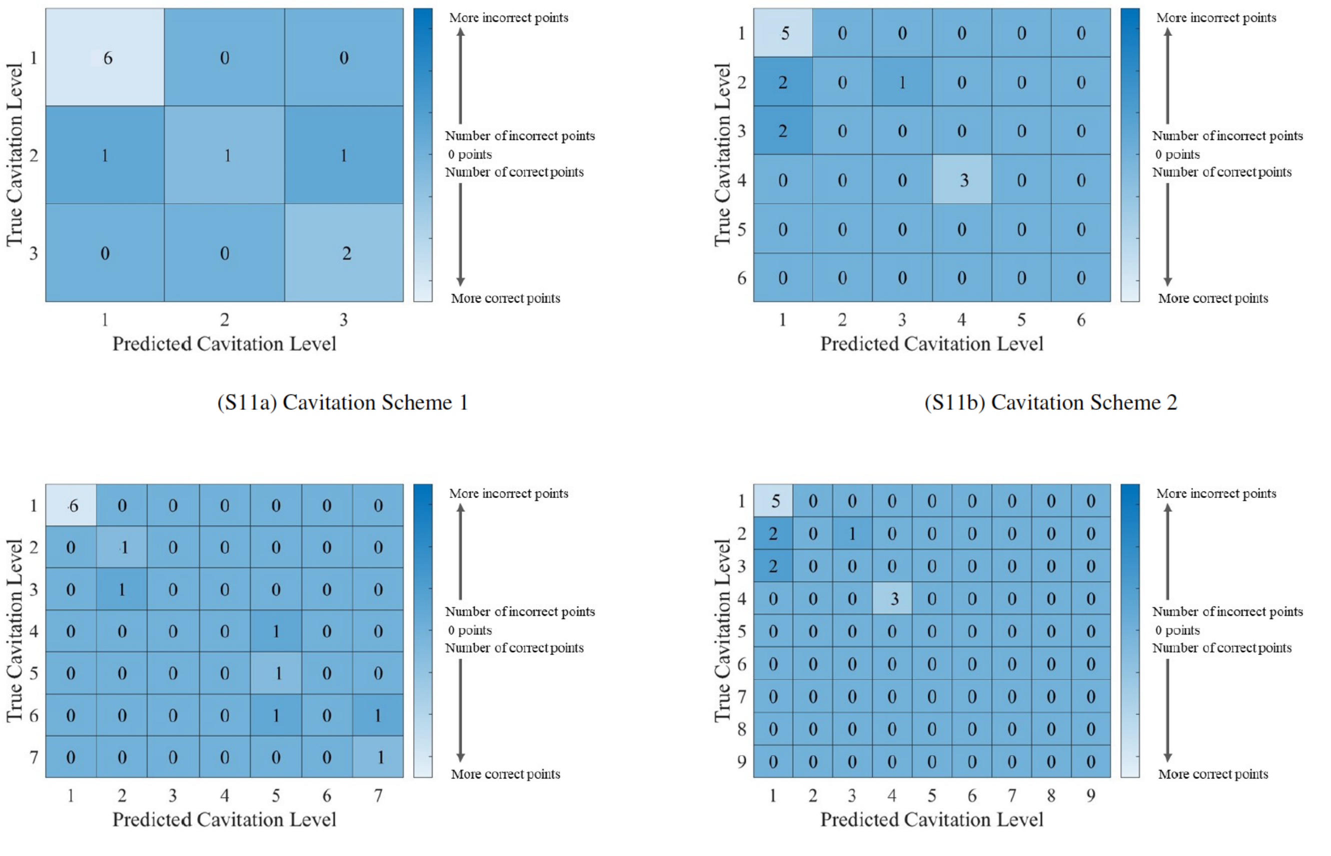

(S11a) Cavitation Scheme 1

(S11b) Cavitation Scheme 2

(S11c) Cavitation Scheme 3

(S11d) Cavitation Scheme 4

Figure S10. Confusion Matrices for the kNN model with $k = 7$ using **(S10a)** cavitation scheme 1, **(S10b)** cavitation scheme 2, **(S10c)** cavitation scheme 3, and **(S10d)** cavitation scheme 4.

2.3 Maximum and Minimum Cross-Validation Accuracy for the kNN Model

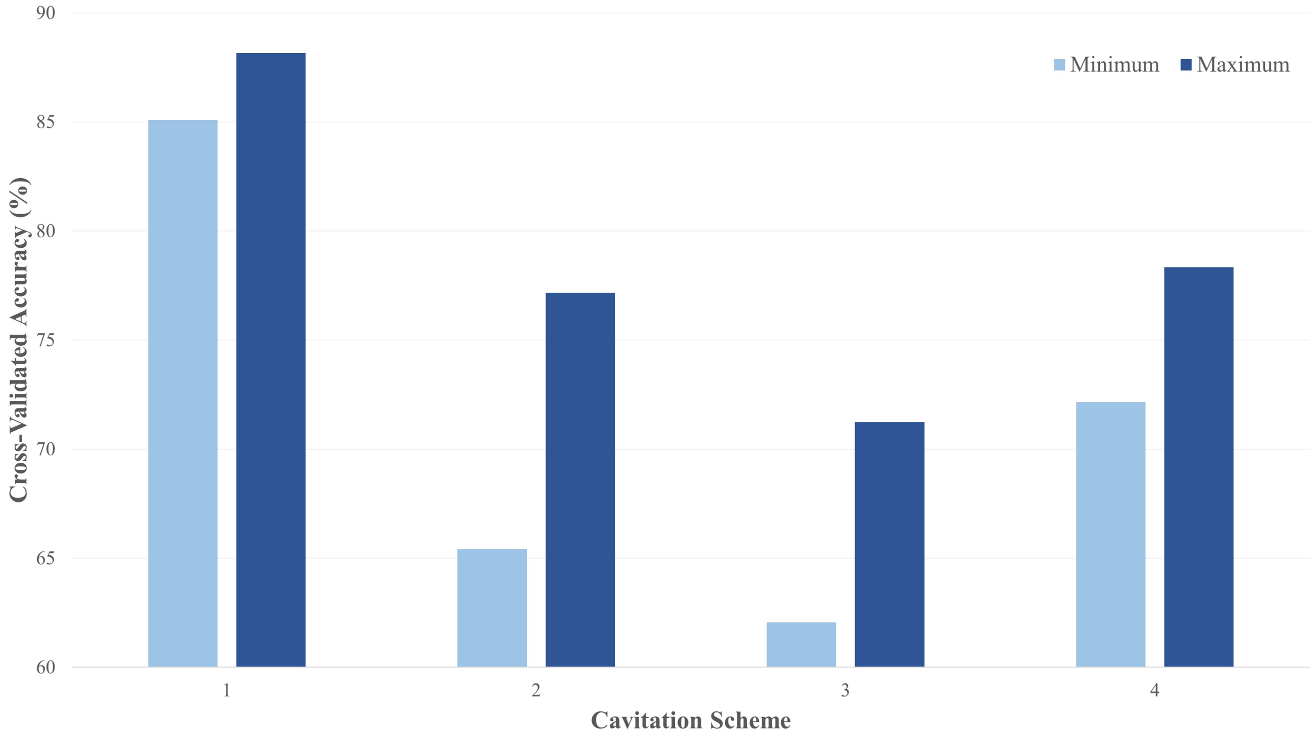

Figure S11. The maximum and minimum cross-validation accuracy attained for each cavitation scheme across all values of k.

2.4 ECOC SVM Model Performance

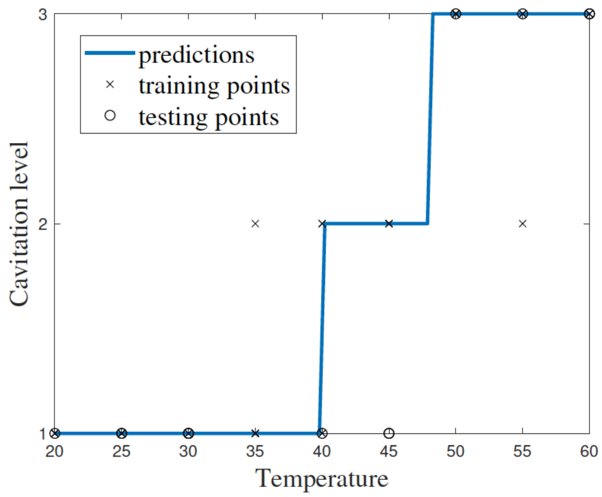

(S6a) Cavitation Scheme 1

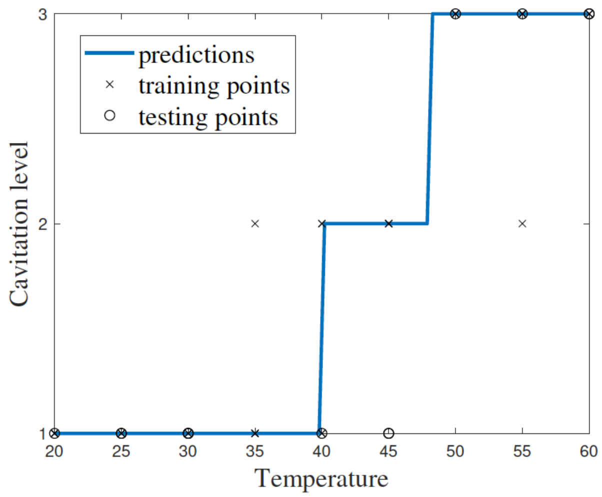

(S6b) Cavitation Scheme 2

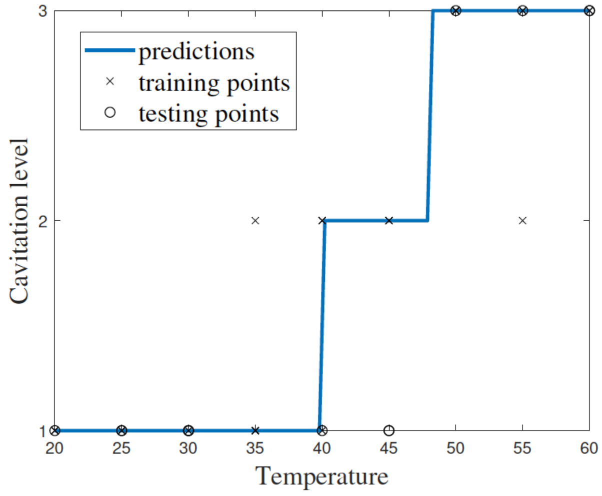

(S6c) Cavitation Scheme 3

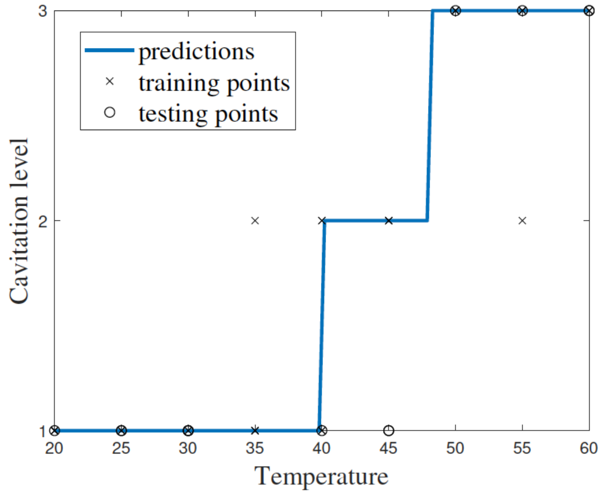

(S6d) Cavitation Scheme 4

Figure S12. ECOC SVM Model Performance for cost $C = 1$ using **(S12a)** cavitation scheme 1, **(S12b)** cavitation scheme 2, **(S12c)** cavitation scheme 3, and **(S12d)** cavitation scheme 4.

2.5 Confusion Matrices for the ECOC SVM Model

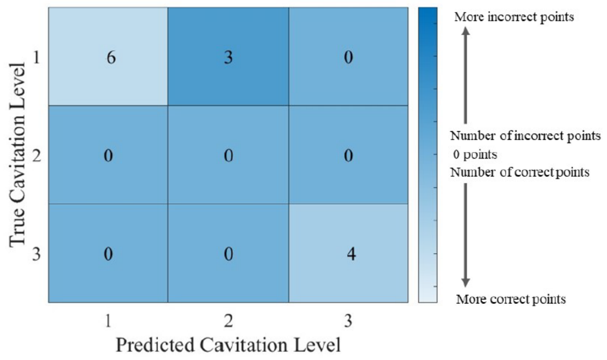

(S12a) Cavitation Scheme 1

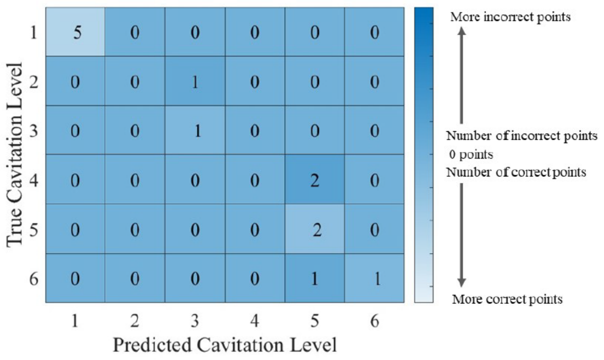

(S12b) Cavitation Scheme 2

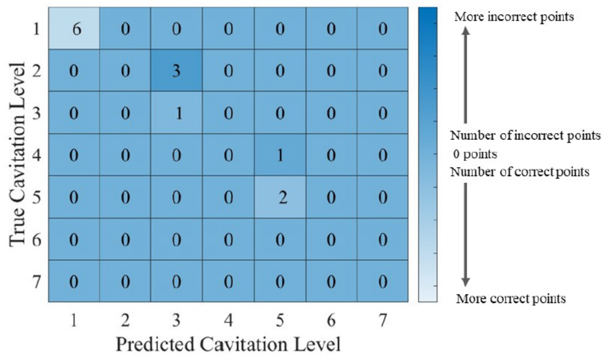

(S12c) Cavitation Scheme 3

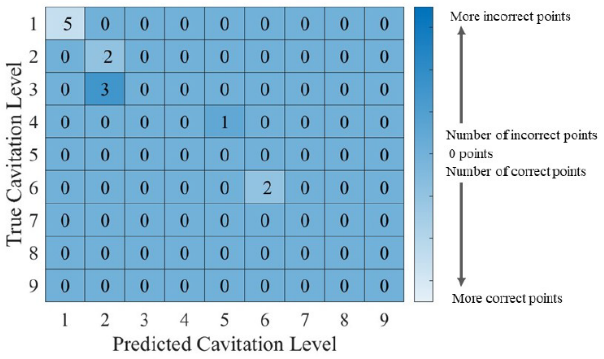

(S12d) Cavitation Scheme 4

Figure S13. Confusion Matrices for the ECOC SVM Model with cost $C = 1$ using **(S13a)** cavitation scheme 1, **(S13b)** cavitation scheme 2, **(S13c)** cavitation scheme 3, and **(S13d)** cavitation scheme 4.

2.6 Bootstrapping Results for the kNN and ECOC SVM Models

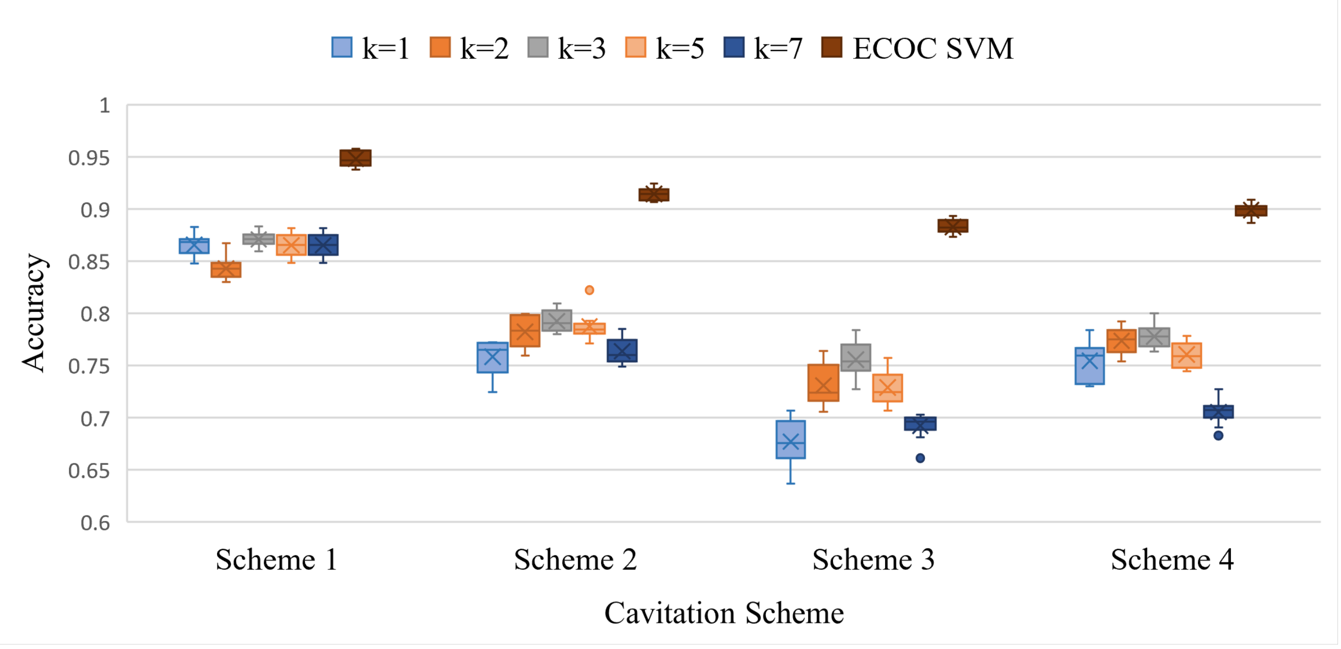

Figure S14. Box-and-Whisker plots of bootstrap sample means for each model (i.e. kNN and ECOC SVM) and cavitation scheme.
